# Development of early life gut resistome and mobilome across gestational ages and microbiota-modifying treatments

**DOI:** 10.1016/j.ebiom.2023.104613

**Published:** 2023-05-13

**Authors:** Ahmed Bargheet, Claus Klingenberg, Eirin Esaiassen, Erik Hjerde, Jorunn Pauline Cavanagh, Johan Bengtsson-Palme, Veronika Kuchařová Pettersen

**Affiliations:** a Host-Microbe Interaction Research Group, Department of Medical Biology, UiT The Arctic University of Norway, Tromsø, Norway; b Paediatric Research Group, Department of Clinical Medicine, UiT The Arctic University of Norway, Tromsø, Norway; c Center for New Antibacterial Strategies, UiT The Arctic University of Norway, Tromsø, Norway; d Department of Paediatrics, University Hospital of North Norway, Tromsø, Norway; e Department of Chemistry, Norstruct, UiT The Arctic University of Norway, Tromsø, Norway; f Division of Systems Biology, Department of Biology and Biological Engineering, Chalmers University of Technology, Gothenburg, SE-412 96, Sweden; g Department of Infectious Diseases, Institute of Biomedicine, The Sahlgrenska Academy, University of Gothenburg, Guldhedsgatan 10A, Gothenburg, SE-413 46, Sweden; h Centre for Antibiotic Resistance Research (CARe), University of Gothenburg, Gothenburg, Sweden

**Keywords:** Extremely preterm infants, Probiotics, Gestational age, Gut microbiota, Resistome, Mobilome

## Abstract

**Background:**

Gestational age (GA) and associated level of gastrointestinal tract maturation are major factors driving the initial gut microbiota composition in preterm infants. Besides, compared to term infants, premature infants often receive antibiotics to treat infections and probiotics to restore optimal gut microbiota. How GA, antibiotics, and probiotics modulate the microbiota’s core characteristics, gut resistome and mobilome, remains nascent.

**Methods:**

We analysed metagenomic data from a longitudinal observational study in six Norwegian neonatal intensive care units to describe the bacterial microbiota of infants of varying GA and receiving different treatments. The cohort consisted of probiotic-supplemented and antibiotic-exposed extremely preterm infants (*n* = 29), antibiotic-exposed very preterm (*n* = 25), antibiotic-unexposed very preterm (*n* = 8), and antibiotic-unexposed full-term (*n* = 10) infants. The stool samples were collected on days of life 7, 28, 120, and 365, and DNA extraction was followed by shotgun metagenome sequencing and bioinformatical analysis.

**Findings:**

The top predictors of microbiota maturation were hospitalisation length and GA. Probiotic administration rendered the gut microbiota and resistome of extremely preterm infants more alike to term infants on day 7 and ameliorated GA-driven loss of microbiota interconnectivity and stability. GA, hospitalisation, and both microbiota-modifying treatments (antibiotics and probiotics) contributed to an elevated carriage of mobile genetic elements in preterm infants compared to term controls. Finally, *Escherichia coli* was associated with the highest number of antibiotic-resistance genes, followed by *Klebsiella pneumoniae* and *Klebsiella aerogenes*.

**Interpretation:**

Prolonged hospitalisation, antibiotics, and probiotic intervention contribute to dynamic alterations in resistome and mobilome, gut microbiota characteristics relevant to infection risk.

**Funding:**

Odd-Berg Group, Northern Norway Regional Health Authority.

**Research in context**

**Evidence before this study**

The gut microbiota composition of preterm infants depends on gestational age (GA) and medical interventions that either disrupt or promote commensal bacteria. However, knowledge gaps remain about the impact of GA and different medical treatments on the infants’ gut microbiota development, including the gut carriage of antibiotic resistance genes and mobile genetic elements.

**Added value of this study**

Using species-level metagenomic data, we showed how supplemented probiotic strains modified the gut microbiota of extremely preterm infants, resulting in a more full-term like gut bacterial community in the first week of life. We also observed that probiotics could diminish the influence of GA by advancing microbiota maturation through enhancing community interconnectedness and stability. Importantly, our findings describe the carriage of antibiotic resistance genes and mobile genetic elements in the gut over the first year of life across infants of different gestational ages, antibiotic exposure, and the absence or presence of probiotic intervention.

**Implications of all the available evidence**

Low GA, combined with prolonged hospitalisation and frequent antibiotic use, negatively alters early life resistome and mobilome, leading to an increased gut carriage of antibiotic resistance genes and mobile genetic elements. The effect of probiotics does not seem to be similarly unidirectional; while decreasing resistome burden, probiotic strains appear to promote mobilome dynamics. Further explorations of these clinically relevant gut microbiota features are necessary to be able to design strategies aiming to lower disease risk in vulnerable preterm infants.

## Introduction

Each year, an estimated 15 million infants are born before 37 weeks of pregnancy are completed, with the global preterm birth rate currently reaching 11% and showing an increasing trend.^1^ Prematurity is a significant cause of morbidity and mortality; approximately 660,000–940,000 children die each year due to complications of preterm birth.^2^^,^^3^ Among severe health complications related to prematurity is an increased risk of infections, partly due to an immature gastrointestinal tract and impaired intestinal barrier function.^4^^,^^5^ Preterm infants are, therefore, often exposed to antibiotics in neonatal intensive care units (NICUs) within the first weeks of life, but this may increase their risk of later short-term complications associated with the gut and lungs.^6^ Antibiotic treatment also depletes symbiotic gut bacteria while enriching the antibiotic resistance gene (ARG) pool, the gut resistome.^7^

Prolonged antibiotic exposures in infancy can have lifelong health implications.^8^ In infants born vaginally and at term, the pioneering gut microbiota confers several essential functions to the developing human physiology, such as educating the immune system, promoting intestinal maturation, and protecting against invading pathogens.^9^ However, premature newborns are disadvantaged compared to term infants, as they display a lower diversity of the gut microbiota, delayed colonisation with founder bacterial species *Bifidobacterium* and *Bacteroides*, and increased abundance of bacteria with pathogenic potentials, such as *Enterobacter*, *Escherichia*, *Enterococcus*, and *Staphylococcus*.^10^, ^11^, ^12^, ^13^ Additionally, preterm infants are often delivered via Caesarean section, which promotes the initial domination of common skin and environmental microorganisms, including *Staphylococcus* and *Propionibacterium*.^14^ Hospitalisation in itself provides an umbrella for several external factors that influence the gut microbiota of preterm infants, including feeding via nasogastric tubes,^15^ formula feeding in the absence of mother’s-own-milk or donor human milk,^16^ parenteral nutrition,^17^ and the microbiota of the health personnel and hospital surfaces.^18^ Many of these factors promote a greater abundance of potential pathogenic taxa mentioned above, while contributing to a low abundance of typical beneficial infant gut bacteria, such as *Bifidobacterium* spp.

Intestinal immaturity, gut microbiota dominated by bacteria with pathogenic potential, and repetitive antibiotic exposure increase the risk of necrotising enterocolitis (NEC) and death in preterm infants.^6^ On the other hand, probiotic supplements containing beneficial bacterial taxa can favourably alter the preterm gut microbiota composition.^19^ Consequently, probiotics are increasingly administered to high-risk preterm infants in NICUs, given their effectiveness in reducing the risk of NEC, sepsis, and death.^20^, ^21^, ^22^ Among the mechanisms of probiotic strains’ action is an enhancement of colonisation resistance, which is the suppression of growth, persistence, and eventual infection by pathogens.^23^ Additionally, probiotics serve as immunobiotics that enhance the immune response and strengthen the epithelial barrier.^24^ However, routine probiotic administration remains controversial because of the risk that preterm infants with an immature immune system could become infected by the supplemented strains^25^ and uncertainty about the effect in the most high-risk preterm infants with a birth weight below 1000 g.^26^

Evidence is scarce regarding how the above microbiota-modifying treatments (antibiotics and probiotics) affect two core characteristics relevant to infection risk: gut resistome and mobilome. In general, neonates display higher gut carriage of ARGs than their mothers,^27^ and among the factors that have been linked to an increased abundance of ARGs are formula feeding,^27^^,^^28^ caesarean section birth,^29^ and maternal antibiotics.^27^ In contrast, probiotic treatment can significantly reduce the gut resistome in preterm infants.^30^ Similarly to resistome, a recent study found that mobile genetic elements (MGEs) are more abundant in newborns than in mothers.^31^ The gut mobilome, the assortment of MGEs, including plasmids, transposons, integrons, and insertion sequences, contributes to the spread of ARGs within gut microbiota.^32^ Preterm infants’ mobilome is associated with hospital location,^33^ as well as their mothers’ mobilome,^27^ which has been recently suggested to shape infant gut microbial assembly and metabolism.^34^ However, there is a paucity of information on the short- and long-term effects of microbiota-modifying treatments on the resistome and mobilome profiles in newborns of different gestational age (GA). Understanding how early life exposure influences the resistome, and mobilome can contribute to strategies that reduce the gut ARGs carriage and, consequently, the incidence of treatment failure due to antibiotic resistance.

Here we report findings from an observational study that included antibiotic- and probiotic-exposed extremely preterm infants (GA < 28 weeks), antibiotic-exposed very preterm infants (GA 28–31 weeks), antibiotic-unexposed very preterm infants (GA 29–31 weeks), and full-term infants, followed from birth up to one year of age. In our previous study describing this cohort,^21^ we detailed the effects of probiotic supplementation and antibiotic therapy on the preterm infants’ gut microbiota and resistome at the genus level. In this study, we performed additional shotgun metagenome sequencing of samples collected at 1 year of life and used innovative computational workflows to evaluate the impact of GA, probiotic supplementation, and antibiotic treatment on the species-level composition, maturation, interconnectivity, and stability of the gut microbiota. We also assessed the influence of GA and the microbiota-modifying treatments on the gut resistome and mobilome, their correlations with the gut microbiota composition, and the co-occurrence of taxa and ARGs. Finally, we performed a strain level analysis for *Escherichia coli*, the species associated with the carriage of the largest number of ARGs, to comprehend its dynamic in the infants’ gut. Our results show a new dimension of the relationship between probiotic supplementation in extremely premature infants and the composition of their gut microbiota, resistome, and mobilome. Such insights are critical for developing clinical strategies aiming at reducing disease risk in this vulnerable population and that promote protective gut microbiota.

## Methods

### Ethics statement

The study was approved by the Norwegian Regional Ethical Committee (2014/930/REK Nord) and registered on Clinicaltrials.gov (https://clinicaltrials.gov/ct2/show/NCT02197468). Informed written consent was obtained from all parents.

### Sample collection

**Fig. 1:**
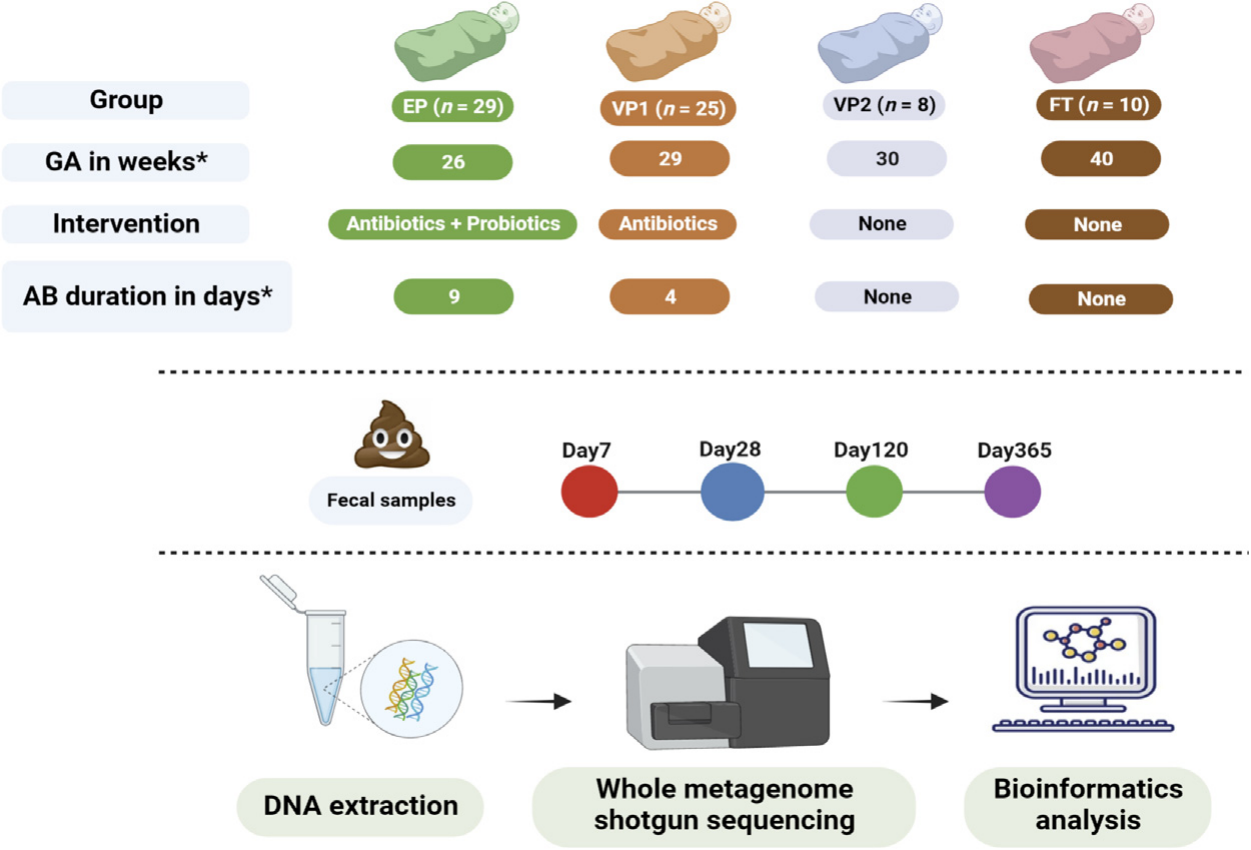
**Study design and sample processing workflow.** Stool samples from extremely preterm (EP), very preterm (VP), and full-term (FT) infants were collected at four indicated time points and followed by DNA extraction, Illumina sequencing, and bioinformatics analyses. The four studied groups differed in gestational age, probiotic supplementation, antibiotic use, and its duration. ∗Median. GA = Gestational age; AB = Antibiotic.

Stool samples were collected in the longitudinal observational study carried out in six neonatal intensive care units (NICUs) in Norway.^21^ We divided the cohort into 4 groups of infants based on probiotic use and antibiotic exposure during their first 4 months of life (Fig. 1 and Table 1): probiotic-supplemented and antibiotic-exposed extremely preterm infants (GA < 28 weeks), antibiotic-exposed very preterm infants (GA 28–31 weeks), antibiotic-unexposed very preterm infants (GA 28–31 weeks), and the control group of healthy term-born antibiotic-unexposed infants. The stool samples were collected at four timepoints (Day 7, 28, 120, 365) and shotgun metagenome sequencing was performed on the extracted DNA. The STORMS checklist^35^ has been completed for concise reporting of the study (Supplementary file S1).

**Table 1:** Clinical characteristics of the infant cohort.

	Extremely preterm (EP) (*n* = 29)	Very preterm 1 (VP1) (*n* = 25)	Very preterm 2 (VP2) (*n* = 8)	Full-term (FT) (*n* = 10)
Birth weight (grams)^a^	807 (675–945)	1235 (1154–1322)	1400 (1225–1538)	3635 (3424–3752)
Gestational age (weeks)^a^	26 (26–27)	29 (28–30)	30 (30–31)	40 (40–41)
Caesarean section/vaginal delivery	20/9	13/12	6/2	0/10
Antibiotic exposure FWL (%)	29 (100)	25 (100)	None	None
Antibiotic exposure after FWL (%)	21 (72)	5 (20)	None	None
Narrow spectrum regimen after FWL (%)	14 (48)	3 (12)	None	None
Broad-spectrum regimen after FWL (%)	8 (28)	2 (8)	None	None
Total days of antibiotics^a^	9 (5–13)	4 (3–6)	None	None
Parenteral nutrition (days)	29	12	1	None
Total days of probiotic supplementation^a^	46 (40–57)	None	None	None
Total days of parenteral nutrition^a^	9 (6–13)	6 (6–8)	3 (3–3)	None
Total days of hospitalisation^a^	80 (11–97)	50 (36–70)	38 (36–43)	2 (1–3)

a Median and interquartile range, FWL = first week of life.

### Probiotic product and administration

The probiotic product in this study was Infloran®; each capsule contained 10^9^ colony-forming units (CFU) of *Bifidobacterium longum* subspecies *infantis* (ATCC 15697) and 10^9^ CFU of *Lactobacillus acidophilus* (ATCC 4356).^21^ From the day of life 3–4, a half-capsule was administered once daily; after 4–7 days, the dosage was doubled to one capsule daily. The content of one capsule was diluted with 2 ml of breast milk or formula and given enterally via an orogastric or nasogastric tube in doses of 1 ml (1/2 capsule) or 2 ml (one capsule).

### DNA isolation and metagenomic library preparation

Total DNA was isolated using the NorDiag Arrow Stool DNA Extraction kit (NorDiag, Oslo, Norway). An additional bead-beating step was implemented to improve cell lysis and boost the extraction of DNA from Gram-positive bacteria.^21^ The extracted DNA was quantified with the Qubit® dsDNA H.R. assay kit (Thermo Fisher Scientific, Waltham, MA, USA) using Qubit® 2.0 Fluorometer (Invitrogen, Carlsbad, CA, USA). DNA purity was analysed using Nanodrop 1000. DNA was stored at −70 °C. The indexed paired-end libraries were produced for whole-genome sequencing using the Nextera XT kit (Illumina, San Diego, CA, USA) according to the manufacturer’s instructions. Genomic DNA (50 ng) was tagmented at 55 °C for 10 min before being amplified with two primers from the Nextera DNA sample preparation Index kit. PCR products were purified using Agencourt AMPure XP beads (Beckman Coulter, Indiana, USA). The purified PCR products were quantified using Qubit® as above. The fragment size distribution (500–1000 bp) was evaluated using the Agilent 2100 Bioanalyzer System (Agilent Technologies, Waldbronn, Germany). Next, the samples were pooled at a concentration of 4 nM per sample. Eight to twelve samples were pooled during each sequencing run, denatured with 0.2 N NaOH, and then diluted to 10 pM with a hybridisation buffer. Finally, samples were submitted for v3 reagents with 2 × 300 cycles paired-end sequencing using the Illumina Miseq platform at the Norwegian Sequencing Centre (Oslo, Norway) with 150-nucleotide-long paired-end reads, according to the manufacturer’s instructions.

### Bioinformatics preprocessing

Before all downstream analyses, pair-end reads were checked for quality using FastQC v.0.11.9. Next, low-quality and adapter sequences were quality-filtered using Trimmomatic v.0.39 with the following parameters: *java -jar trimmomatic-0.39.jar P.E. -phred33 ILLUMINACLIP: NexteraPE-PE.fa:2:30:10 LEADING:3 TRAILING:3 SLIDINGWINDOW:4:15 MINLEN:36*. Trimmed sequences were again assessed for their quality by FastQC v.0.11.9 before further analysis. The human DNA contaminant sequences were discarded from all samples by filtering out the reads mapped against the human reference genome (GRch38, downloaded from NCBI GenBank) using Bowtie2 v.2.4.4 with -*-very-sensitive* parameter. The identified paired reads that did not map against the human genome using SAMtools v.1.12 with *-f 12 -F 256* were used in subsequent analyses.

### Metagenome profiling

High-quality reads were subjected to the CHOCOPhlAn database using MetaPhlAn3 v.3.0.7^36^ to determine the relative abundances of microbial taxa. The *‘merge metaphlan tables.py’* script was used to merge relative abundance tables. The quality-filtered short-read sequences were assembled into longer contiguous sequences (contigs) using metaSPAdes v.3.15.0 with the default parameters. For the assessment of assemblies performed by metaSPAdes, MetaQUAST from QUAST v.5.0.2 was used with the *-m 1000* option (Supplementary Table S1). Open reading frames (ORFs) were predicted from the assembled contigs using Prokka v.1.14.5, with *--kingdom Bacteria --centre X --compliant --mincontig 200* options. Next, redundant ORFs were collapsed to one sequence using CD-HIT v.4.8.1, with the following parameters: *-M 0 -T 0 -c 0.95 -n 8*. The ORFs were annotated by searching nucleotide sequences against the Comprehensive Antibiotic Resistance Database (CARD v.3.0.9)^37^ using ABRicate v.1.0.1 with *--minid 80 --mincov 80* parameters. To estimate the abundance of the annotated ARGs, all fasta files (with nucleotide sequences) generated by Prokka were concatenated in one file. From this, ARGs were extracted from the file using Seqtk v.1.3. Next, redundant ARGs were clustered by CD-HIT v.4.8.1, with the following parameters: *-M 0 -T 0 -c 0.80 -n 8* to create a custom database. Metagenomic reads were mapped against the custom database using Bowtie2 v.2.4.4 with the parameter *--very-sensitive-local* to perform resistome annotation. Using SAMtools v.1.12, mapped reads were separated from unmapped reads, sorted, and indexed, and the number of reads mapped for each ARG was calculated. The counts were then normalised for each sample to the total gene lengths by calculating reads per kilobase reference per million reads (RPKM). The normalised relative abundances of MGEs were evaluated by processing the quality-controlled FASTQ files with ShortBRED v.0.9.3 using a reference database of MGEs (transposases, integrases, recombinases, and integrons) curated by NanoARG v.1.0.^38^ Employing HUMAnN v. 3.0.1,^36^ metabolic pathway abundances were estimated. Using the utility scripts *“humann2 renorm table”*, *“humann2 regroup table”*, and *“humann2 join tables”*, raw count data were normalised for sequencing depth, compressed by ontology, and tables were merged. Strain analysis was performed using the StrainGST from StrainGE v. 1.3.3^39^ upon downloading the NCBI reference genome for *E. coli*.

### Statistical analysis and data visualisation

The statistical analysis was carried out using R v.4.1.2. For analysis of variance, the *aov* function was applied, followed by a post hoc correction for multiple comparisons using the Tukey HSD (honestly significant difference) test. *LongDat* R package v.1.0.3 was used to determine if there were significant changes in features over time and any potential batch effect resulting from processing the samples in two sequencing runs (Supplementary Fig. S1). We used the *vegan* v.2.5.7^40^ and *phyloseq* v.1.38.0^41^ packages for diversity analyses (α- and β-diversity). The β-diversity was conducted on the microbiota and resistome data using the function ‘*vegdist*’ of the *vegan* package with principal coordinate analysis (PCoA) and Bray–Curtis dissimilarity indexes. Permutational multivariate analysis of variance (PERMANOVA) was performed using the function ‘*adonis*’ of the *vegan* package with 9999 permutations to determine the statistical significance of composition differences.

Differentially abundant features between time points within treatment groups at family, genera, and species levels were identified using the DESeq2 v.1.34.0 package^42^ with the *p* values attained by the Wald test were corrected for multiple testing using the Benjamini Hochberg method. Procrustes analysis was performed to assess the correlation between taxonomic composition, resistome, and mobilome to comprehend whether any of them was responsible for the other structure. PCoA ordinations of ARG and taxonomic compositions were uniformly scaled and rotated until their squared differences were minimised.^43^ The symmetric Procrustes correlation coefficients and statistics were conducted using the ‘*procrustes*’ and ‘*protest*’ functions from the *vegan* package with 9999 permutations.

Enterotyping analysis was conducted using Dirichlet multinomial mixture (DMM) model using the *DirichletMultinomial* v. 1.36.0 package.^44^ Random forest analysis was conducted using *randomForest* v.4.7.1.1^45^ and caret v. 6.0.93^46^ packages, using 10-fold cross-validation, 500 trees, and 1000 permutations. Microbiota network analysis was carried out independently for each cluster and at the genus level. Genera with less than 25% prevalence were excluded. We performed a centre log-ratio transformation using the *composition* package v.2.0.4, allowing us to apply conventional statistical techniques to the transformed compositional data. Next, using the *Hmisc* package v.4.7.0, partial correlations were analysed using Spearman rank correlations, and correlations with absolute coefficients of more than 0.20 were represented as networks using the *qgraph* package v. 1.9.2. The probabilities of Markov chain state transitions were calculated with the *markovchain* package v.0.8.6 and illustrated with *DiagrammeR* package v.1.0.9. We performed the co-occurrence analysis by constructing a correlation matrix based on pairwise Spearman’s correlations among microbiota and ARG using the *Hmisc* package v.4.7.0. The correlation was considered statistically robust as the Spearman correlation coefficient was more than 0.8 and the *p*-value was less than 0.01.^47^ Clinical differences between groups were assessed using parametric or non-parametric tests, as appropriate. *p*-values less than 0.05 were considered significant. The graphical illustrations were created with the *ggplot2* v.3.3.6 package with post-editing in Adobe Illustrator.

### Role of the funding source

The funders had no role in the study design, data collection, data Formal analysis, interpretation, or report writing.

## Results

### Cohort description

The cohort in this study included 29 extremely preterm infants (EP) and 33 very preterm infants (VP) (Fig. 1). EP infants received probiotic supplementation and were treated with antibiotics shortly after birth (Table 1). Among VP infants, one group received antibiotic therapy (VP1; *n* = 25), while another group was not exposed to antibiotics (VP2; *n* = 8). This allowed us to utilise VP2 as a GA-matched control for VP1. EP infants received antibiotics for longer time than VP1 infants (median 9 days vs 4 days, respectively). The EP group also had a significantly longer hospital stay than VP infants (Wilcoxon rank-sum; *p* < 0.01) and there was a moderate correlation between longer hospital stay and lower GA for individual infants (Spearman’s rho: −0.45, *p* < 0.01). Two infants belonging to the EP group developed NEC (Supplementary Table S2) and underwent surgical intervention, but none from the preterm infant groups (EP, VP1, or VP2) progressed towards sepsis. Finally, we included 10 antibiotic-unexposed, healthy, vaginally delivered full-term (FT) infants.

### Impact of probiotic supplementation on microbiota composition, diversity, and functional profile

**Fig. 2:**
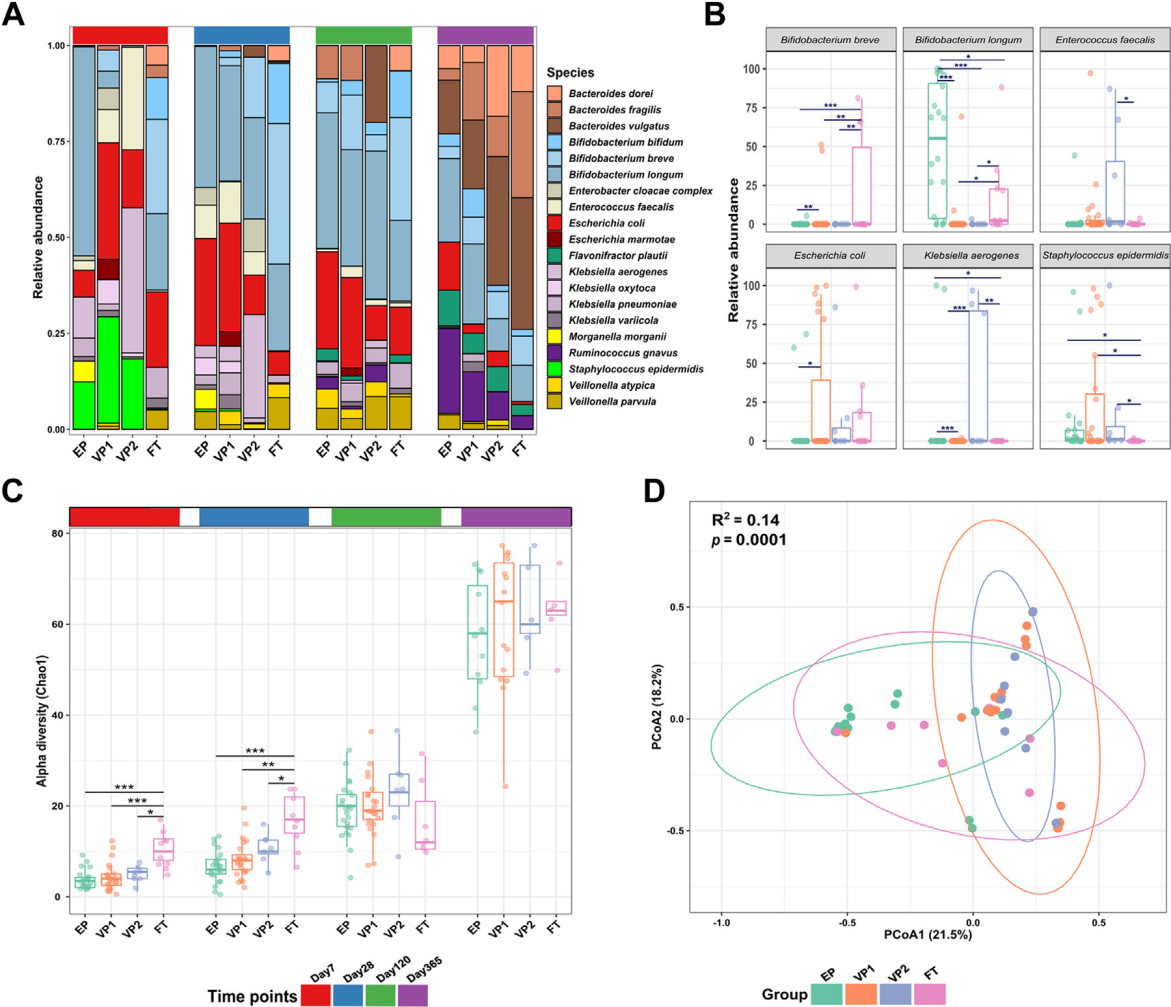
**Bacterial microbiota of infants with varying gestational age and receiving different microbiota-modifying treatments. (A)** The relative abundance of 20 most abundant bacterial species in extremely preterm (EP = antibiotic-exposed and probiotic supplemented), very preterm (VP1 = antibiotic-exposed, VP2 = antibiotic naive), and full-term infants (FT = antibiotic naive), as inferred by MetaPhlAn3. **(B)** Despite large interindividual variability, the relative abundances of several bacterial species showed significant differences across groups on day 7, as estimated by DESeq2. The *p* values were computed using the Wald test. Adjusted *p* values (adj. *p*): ∗∗∗adj. *p* < 0.001; ∗∗adj. *p* < 0.01; ∗adj. *p* < 0.05. **(C)** Chao1 diversity comparison between the groups. Each point represents a sample. The horizontal box lines represent the first quartile, the median, and the third quartile. The *p* values were computed using the One-way ANOVA and adjusted using Tukey’s HSD post hoc test. Adjusted *p* values (adj. *p*): ∗∗∗adj. *p* < 0.001; ∗∗adj. *p* < 0.01; ∗adj. *p* < 0.05. **(D)** Principal coordinate analysis (PCoA) based on the Bray–Curtis dissimilarity matrix and PERMANOVA test showed a significant shift in the microbiota composition on day 7. Except for VP1 vs VP2 (*p* = 0.09), the microbiota composition in each group was significantly dissimilar. Each point represents the bacterial microbiota of an individual sample. Ellipses represent a 95-confidence interval.

The probiotic supplementation immediately affected the gut bacterial composition of EP infants. On day 7, and compared to the VP groups, EP infants had a significantly lower relative abundance of potentially pathogenic bacteria, namely, *E. coli*, *Enterococcus faecalis*, *Klebsiella aerogenes*, and *Staphylococcus epidermis* (Fig. 2A). Although we observed large variation between individual infants, the relative abundance of *B. longum* in EP infants was significantly higher than in the other groups on day 7 (Wald; EP vs VP1 adj. *p* < 0.001; EP vs VP2 adj. *p* < 0.001; EP vs FT adj. *p* = 0.014) while FT infants were dominated by *Bifidobacterium breve* (Fig. 2B). On day 28, the relative abundance of *B. longum* in the VP groups increased, and we found no significant differences in the relative abundances of *B. longum* between the groups from this point and up to 12 months of age. The species-level differences were also reflected on the bacterial genus level (Supplementary Fig. S2).

We next evaluated the alpha and beta diversity of the infants’ gut microbiota. Across all groups, Chao1 and Shannon diversity indices increased over time. On days 7 and 28, the microbiota richness (Chao1) in the FT control group was significantly higher than for the groups born preterm (Fig. 2C), and we found no significant differences between preterm infants’ groups throughout the study. We also performed ordination analysis to identify similarities between samples using the relative abundance of microbiota at the species level. The microbial communities of the different infant groups were significantly dissimilar from each other on day 7 (PERMANOVA: Bray–Curtis distance; *p* < 0.001) apart from VP1 and VP2 (PERMANOVA: Bray–Curtis distance; *p* = 0.094), which were more similar to each other than to the other groups (Fig. 2D). On day 28, the microbial communities were also dissimilar between EP and FT (PERMANOVA: Bray–Curtis distance; *p* = 0.0001) and VP1 and FT (PERMANOVA: Bray–Curtis distance; *p* = 0.002). We found no statistical differences in beta diversity over other time points. Lastly, delivery mode impacts the composition of the gut microbiota in full-term newborns,^48^ prompting us to explore potential differences across the groups of similar GA but born either via Caesarean section or vaginally. The analyses revealed no statistically significant differences within the EP and VP groups (Supplementary Fig. S3).

Besides the observed variations in the gut microbiota composition, we observed relative functional stability of the infants’ gut microbiota over time, as predicted by HUMAnN3. An exception was the EP group on day 7, which displayed a significantly lower Shannon diversity index for metabolic pathways than the other infant groups (Supplementary Fig. S4).

### Probiotic supplementation stimulates gut microbiota development in extremely preterm infants

**Fig. 3:**
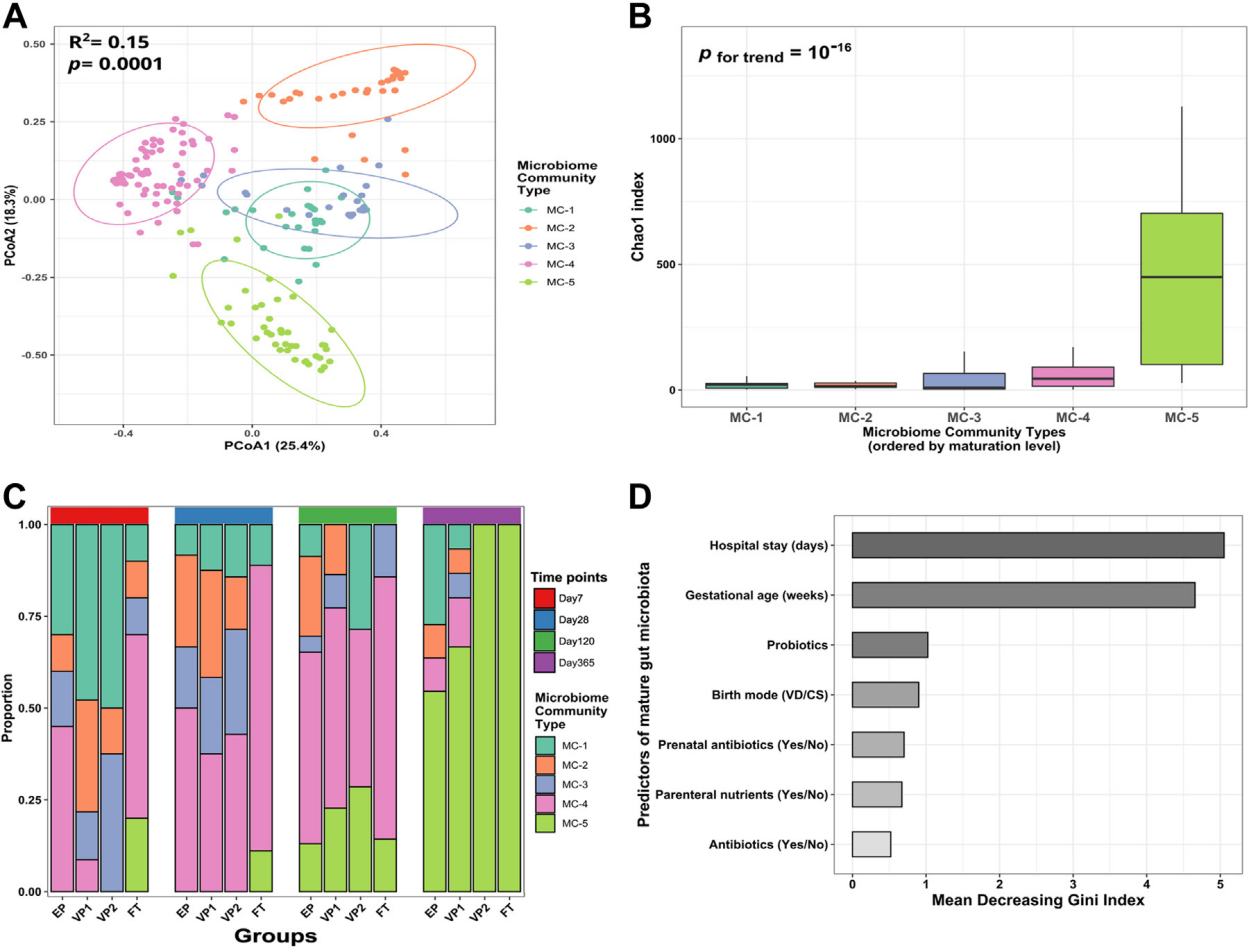
**The gut microbiota maturation of infants with varying gestational ages and receiving different microbiota-modifying treatments. (A)** Five gut microbial community (MC) types were identified using the Dirichlet Multinomial Mixture modelling applied to all study samples. The Bray–Curtis dissimilarity matrix and PERMANOVA were used to investigate and test the association of MC types with beta diversity. Each point represents a sample, and the ellipses represent a 95-confidence interval. **(B)** Comparison of the MC types richness (Chao1). The horizontal box lines represent the first quartile, the median, and the third quartile **(C)**. The distribution of MC types across the infant groups (extremely preterm EP - antibiotic-exposed and probiotic supplemented; very preterm VP1 - antibiotic-exposed and VP2 - antibiotic unexposed; full-term infants FT - antibiotic unexposed) and four time points. **(D)** Predictors of mature MC type (MC-4 and MC-5 vs MC-1, MC-2, and MC-3 combined) ranked by their relevance as determined by random-forest modelling using 1000 permutations and 500 trees. We categorised MC-4 and MC-5, enriched in *Bifidobacterium* and *Bacteroides*, respectively (Supplementary Fig. S5), as mature MC types because they emerged in full-term infants on day 7.

We next evaluated the patterns of gut microbiota assembly across the infant groups with different GA and interventions. Employing an unsupervised machine learning approach based on Dirichlet multinomial mixtures for probabilistic data clustering, we identified five microbial community (MC) types (Fig. 3A). MC-1 was dominated by *Staphylococcus*, while MC-4 and MC-5 were enriched in *Bifidobacterium* and *Bacteroides*, respectively (Supplementary Fig. S5). Compared to other MC types, MC-5 had the highest alpha diversity (Fig. 3B) and was identified in the FT infants on days 7 and 28 (Fig. 3C), indicating a progressive maturation of the MC types with age. Based on the early emergence of *Bifidobacterium*-enriched MC-4, and *Bacteroides*-enriched MC5 types in FT infants, we categorised them as the most matured MC types compared to MC-1 to 3.

MC-1 dominated on day 7 in the VP groups, predicting the influence of low GA on microbiota maturity. Even though EP infants were expected to have less mature MC types due to the GA effect, supplementation with the probiotic strains resulted in EP infants displaying MC-4 on day 7. However, at later time points (days 28 and 120), the MC maturation level for VP infants became comparable to EP infants. At 1 year of life, the EP and VP1 groups still exhibited MC-1 and MC-2 compared to the antibiotic-unexposed groups VP2 and FT infants, who displayed exclusively MC-5 (Fig. 3C).

To investigate the drivers of microbiota maturation in preterm infants, we ran a random-forest classifier to determine factors that predict maturation towards MC-4 and MC-5 types and their relative importance (Fig. 3D). The FT group was excluded from this analysis. Aside from GA and length of hospitalisation, which were the primary predictors, probiotic supplementation was the third most important variable predicting intestinal microbiota maturation status. The decreasing Gini index further indicated more contribution of the probiotic intervention to the gut microbiota maturation in EP infants than other measured external factors such as antibiotic treatment and parenteral nutrients.

### Probiotics promote a microbial community with greater interconnectivity and stability

**Fig. 4:**
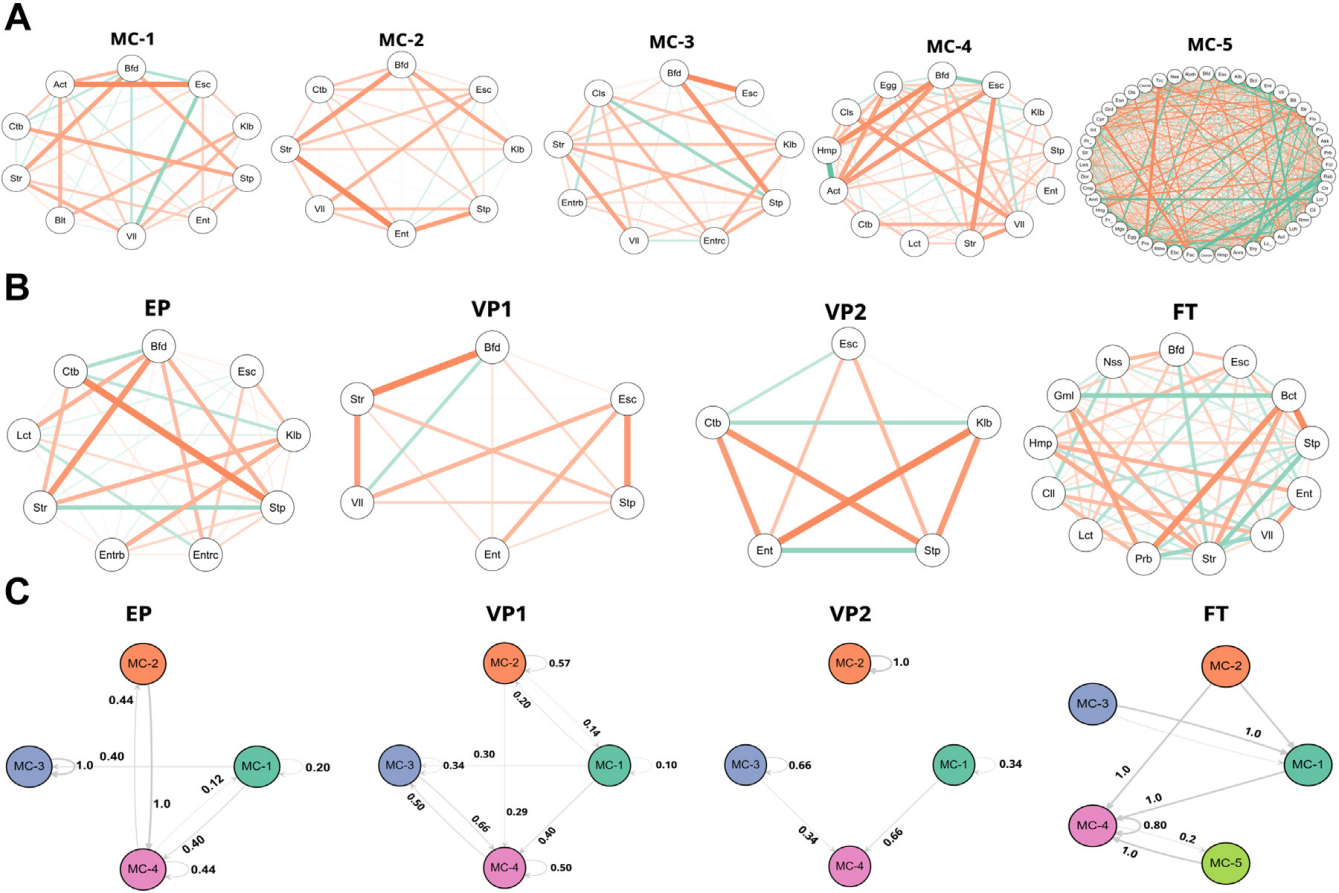
**Early life microbial community interconnectivity and stability.** Network analysis along the microbiota maturation trajectory **(A)** and different infant groups at day 7 **(B)**. Each node represents one bacterial genus, and the connection represents Spearman’s correlation coefficient. The greater the Spearman’s correlation coefficient, the thicker the line connecting the genera. Aquamarine colour exhibits a positive correlation, whereas tangerine indicates a negative correlation. **(C)** Stability and the likelihood of transitioning amongst the microbial community (MC) types were assessed by Markov Chain modelling and compared in the different infant groups. Only the first two time points, covering the window of probiotic intervention, were considered in the analysis. Each node represents an MC type identified by the Dirichlet Multinomial Mixture model. Abbreviations: extremely preterm infants (EP), very preterm infants (VP), and full-term infants (FT). Abbreviations of bacterial genera can be found in Supplementary Table S5.

We next evaluated how the different external factors shape the gut microbiota by determining interconnectivity, stability, and the probability of transition across MC types. The network analysis on day 7 revealed that the interconnectivity was highest for the most mature MC-4 and MC-5 types (Fig. 4A). The community interconnectivity was highly influenced by GA with increased community connectedness in FT infants compared to the premature infants (Fig. 4B). However, the probiotic supplementation seemed to diminish the GA influence and enhance the interconnectivity of the microbial community. Interestingly, when comparing VP1 and VP2, antibiotic therapy had little or no influence on microbial community interconnectivity. We found subtle differences in the interconnectivity across the preterm infants on day 28 and no differences between groups at the remaining time points.

Finally, we included a Markov chain analysis to estimate the likelihood of transitions between the community types using the first two time points, covering the window of probiotic intervention, for assessing the influence of the probiotics on the probability transition from less matured to more matured community type during that period. This statistical modelling suggested that the probiotic supplementation increased the probability of both maturing to MC-4 and remaining at MC-4 (Fig. 4C). By comparing the VP1 and VP2 groups, antibiotic treatment had a negligible impact on the transition probabilities to a less developed microbial community. In contrast, the FT group showed a much higher chance of transitioning from less matured communities and eventually staying in the more matured ones compared to the other groups, implying that GA strongly influences gut microbiota interconnectivity and stability.

### Hospitalisation and probiotics supplementation modulate the gut resistome burden

**Fig. 5:**
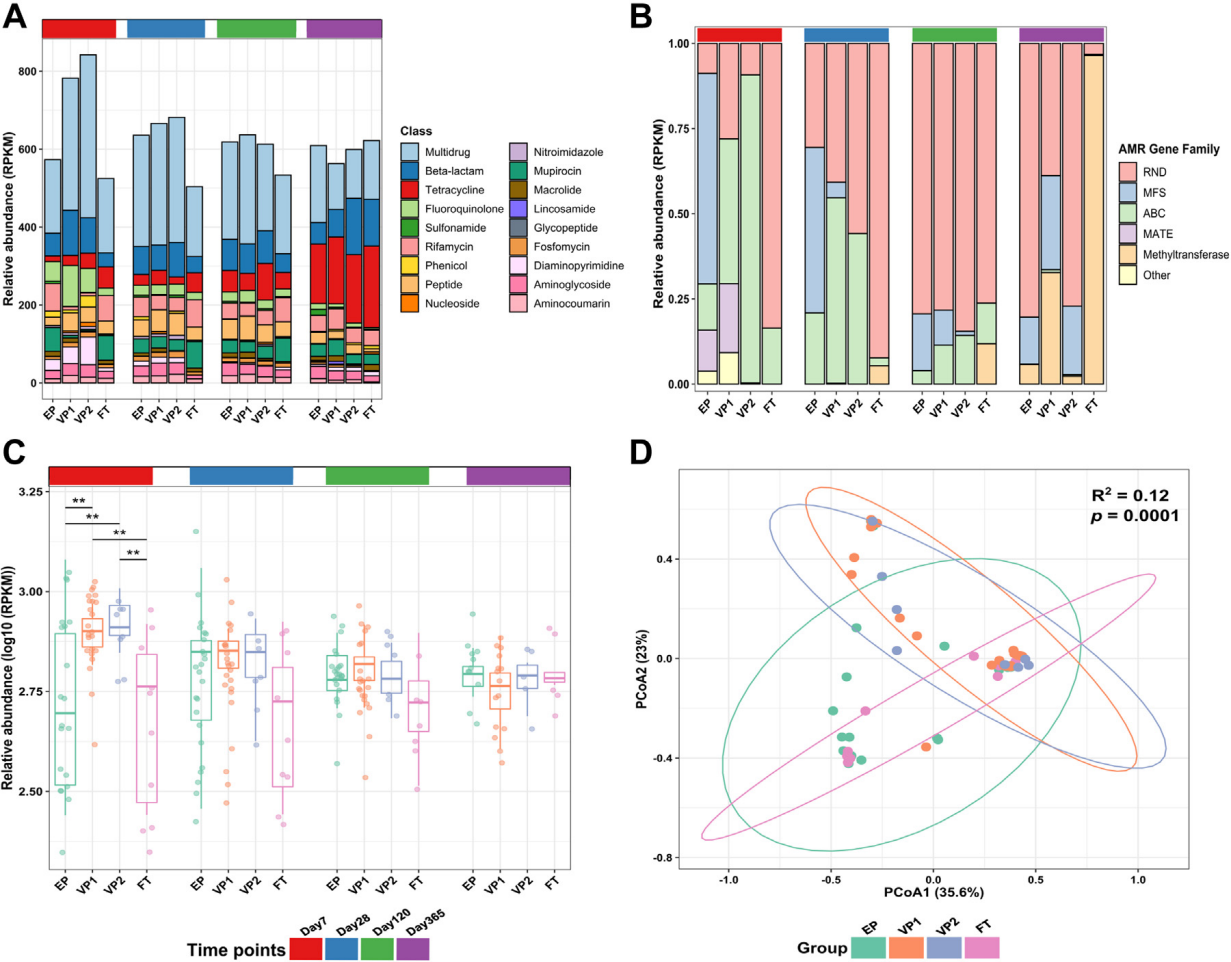
**Resistome composition across different infant groups. (A)** Relative abundance of antibiotic resistance genes (ARGs) in reads per kilobase per million mapped reads (RPKM) stratified by antibiotic classes. **(B)** Relative abundance of genes in RPKM that confer multidrug resistance (MDR) grouped by antimicrobial-resistant gene families. **(C)** Log_10_ of the relative abundance of ARGs in RPKM. Each point represents a sample. The horizontal box lines represent the first quartile, the median, and the third quartile. The *p* values were computed using the One-way ANOVA and adjusted using Tukey’s HSD post hoc test (∗∗adj. *p* < 0.01). **(D)** The Bray–Curtis dissimilarity matrix and PERMANOVA test showed a significant shift in the resistome composition on the day 7 timepoint. Except for infant group comparisons VP1 vs VP2 and EP vs FT (*p* = 0.423, and *p* = 0.337), the resistome composition in each group was significantly dissimilar from any other group. Each point represents the resistome of an individual sample. Ellipses represent 95 confidence intervals. Abbreviations: extremely preterm infants (EP), very preterm infants (VP), full-term infants (FT), resistance-nodulation-division (RND), major facilitator superfamily (MFS), ATP binding cassette (ABC), and Multidrug And Toxic compound Extrusion (MATE).

The antibiotic resistome is a core characteristic of the infant gut microbiota,^32^ and the resistome burden correlates with cumulative antibiotic exposure.^7^ To discern the effects of probiotic supplementation, antibiotic exposure, and differences in GA on the resistome dynamics, we searched the metagenomic data against the CARD database and analysed the resulting resistome profiles independently of the microbiota composition. We detected 209 unique ARGs conferring resistance to 21 antibiotic classes (Supplementary Table S3). The dominating ARGs in all infant groups were genes linked to multidrug resistance (Fig. 5A). Initially, we tested for differences in the preterm groups’ resistome profiles based on the birth mode, since birth mode has been reported to influence the full-term infant resistome.^29^ However, we found no significant difference (Supplementary Fig. S6).

GA and associated external factors appeared to strongly influence the ARGs repertoire on day 7. ARGs from three families of multidrug resistance efflux pumps; the major facilitator superfamily (MFS), the ATP binding cassette (ABC), and transporters in the resistance-nodulation-division (RND), comprised the majority of ARGs in the EP, VP, and FT groups, respectively (Fig. 5B). At days 28 and 120, the RND family became the most dominant throughout the samples. At 1 year of life, the EP and VP2 groups were still dominated by ARGs from the RND family compared to FT infants, who displayed almost exclusively ARGs from the methyltransferase class.

On day 7, the gut microbiota of EP infants had significantly lower relative abundances of ARGs than the VP groups (Tukey’s HSD; EP vs VP1 adj. *p* = 0.004, EP vs VP2 adj. *p* = 0.008). The ARG levels of the FT group were almost identical to EP infants (Tukey’s HSD; adj. *p* = 0.79) and significantly lower than VP (Tukey’s HSD; FT vs VP1 adj. *p* = 0.004, FT vs VP2 adj. *p* = 0.005). Interestingly, the antibiotic-treated VP1 group did not show a significant difference in the ARG relative abundance compared to VP2 group that had not received antibiotics. There were no significant differences between the groups throughout the other time points, although the FT infants displayed a trend of lower ARG abundances on days 28 and 120 (Fig. 5C).

We next evaluated differences in the groups’ resistome profiles at the gene level. On day 7, the relative abundance of genes conferring resistance to β-lactams was significantly higher in the VP1 group than in the EP (Wald; adj. *p* = 0.025) and FT (Wald; adj. *p* = 0.0005) group (Supplementary Fig. S7). Moreover, the relative abundance of fluoroquinolone ARGs was higher in the VP1 group than what was identified for the other groups; however, the difference was only significant in comparison to the FT group (Wald; *p* = 0.00031). Additionally, ARGs that confer multidrug resistance in the VP groups were significantly higher than the EP and FT infants (Wald; adj. p = 0.025 and adj. *p* = 0.035), respectively. We observed no significant differences between the groups throughout the other time points.

Finally, we investigated resistome diversity to evaluate ARGs diversification over different GA and treatments. Overall, both Chao1 and Shannon indices showed no significant differences in the ARGs diversity among the infant groups (Supplementary Fig. S8). Still, the ARG composition of the infant groups was significantly dissimilar on day 7 (PERMANOVA: Bray–Curtis distance; *p* = 0.0001), except between the infants’ groups VP1 vs VP2 (PERMANOVA: Bray–Curtis distance; *p* = 0.423) and EP vs FT (PERMANOVA: Bray–Curtis distance; *p* = 0.337) (Fig. 5D). Resistome of the EP group differed significantly from VP infants (PERMANOVA: Bray–Curtis distance; EP vs VP1 *p* = 0.0012, EP vs VP2 *p* = 0.0167), similarly to the resistome of FT infants (PERMANOVA: Bray–Curtis distance; FT vs VP1 *p* = 0.0102, FT vs VP2 *p* = 0.0292).

### Development of the gut mobilome across different gestational ages and early life treatments

**Fig. 6:**
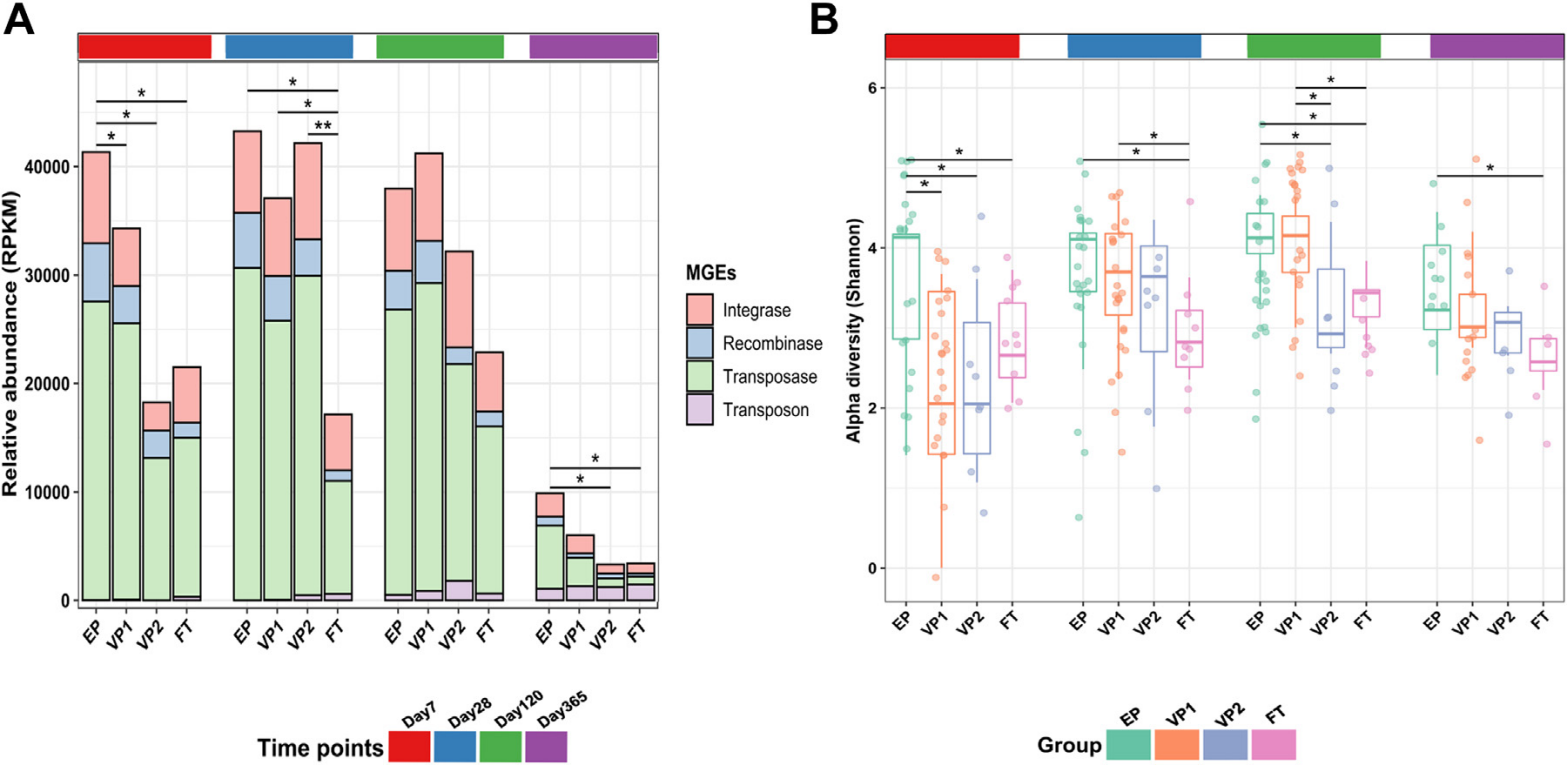
**Mobile genetic elements (MGE) composition across different infant groups. (A)** Relative abundance of MGEs in reads per kilobase per million mapped reads (RPKM). The relative abundance of the MGEs identified on day 7 in the EP infants was significantly higher than those identified for the other groups (Tukey’s HSD post hoc test; EP vs VP1 adj. *p* = 0.031; EP vs VP2 adj. *p* = 0.029; EP vs FT adj. *p* = 0.036). On day 28, the FT group had significantly lower MGEs abundance than the other groups (FT vs EP adj. *p* = 0.003; FT vs VP1 adj. *p* = 0.035; FT vs VP2 adj. *p* = 0.031). On day 120, there were no significant differences between groups. The MGEs levels plunged on day 365, with the significance between EP vs VP2 (adj. *p* = 0.031) and EP vs FT (adj. *p* = 0.033). **(B)** Shannon evenness index comparison between the groups. Each point represents a sample. The horizontal box lines represent the first quartile, the median, and the third Quartile. The *p* values were computed using the One-way ANOVA and adjusted using Tukey’s HSD post hoc test (∗adj. *p* < 0.05). Abbreviations: extremely preterm infants (EP), very preterm infants (VP), and full-term infants (FT).

Mobile genetic elements may contribute to the spread of ARG through horizontal gene transfer between bacterial strains. To investigate the potential for transmission of ARGs, we evaluated the mobilome profiles using ShortBRED in conjunction with a curated database of transposases, integrases, recombinases, and integrons.^38^ We identified all these MGEs classes (Fig. 6A), with most sequencing reads being assigned to ShortBRED markers for the transposase family. This family of enzymes binds to the ends of transposons and, in that way, facilitates DNA transfer from one region of the genome to another via a cut-and-paste mechanism.^49^

We observed distinct trends of mobilome development across the infant groups. The relative abundance of the MGEs identified on day 7 in the EP group was significantly higher than those identified for any other group (Tukey’s HSD; EP vs VP1 adj. *p* = 0.031; EP vs VP2 adj. *p* = 0.029; EP vs FT adj. *p* = 0.036) (Fig. 6A). Furthermore, the MGE levels in the EP and VP groups differed significantly from the FT group on day 28 (Tukey’s HSD; FT vs EP adj. *p* = 0.003; FT vs VP1 adj. *p* = 0.035; FT vs VP2 adj. *p* = 0.031). On day 120, there were no significant differences between groups. In all groups, the relative abundances of MGEs declined gradually towards day 365, with significantly lower levels in FT than in the EP group (Tukey’s HSD; adj. *p* = 0.033) and in VP2 infants (Tukey’s HSD; adj. *p* = 0.031).

We then performed a diversity analysis to evaluate how MGEs were distributed across the samples. On day 7, the Shannon diversity of MGEs in the EP group was significantly greater than for the other groups (Tukey’s HSD; EP vs VP1 adj. *p* = 0.002; EP vs VP2 adj. *p* = 0.032; EP vs FT adj. *p* = 0.026) (Fig. 6B). On days 28 and 120, the Shannon diversity for the FT group was significantly lower than for the antibiotic-treated preterm infants (EP and VP1) (Tukey’s HSD; adj. *p* < 0.05) and remained significantly lower than for the EP group at 1 year of life (Tukey’s HSD; adj. *p* = 0.031). Given these surprising results, we screened the Infloran® probiotic strains for ARGs and MGEs to clarify whether supplementation with the probiotic product might have influenced the propagation of ARGs. Although we found no ARGs encoded in the genomes of the strains, we identified MGEs, with transposases constituting about 55% of the total identified MGEs classes in Infloran® (Supplementary Fig. S9).

### Correlation between microbiota, ARG, and MGE composition

After describing the different microbiota characteristics, we asked to what extent the bacterial community structure shapes the ARG and MGE profiles. The Procrustes analysis identified statistically significant associations between taxonomic, ARG, and MGE composition in the four groups on day 7 but not at other time points (Monte Carlo permutation; *p* < 0.01) (Supplementary Fig. S10). Nevertheless, the correlation between ARGs and MGEs remained significant at the subsequent time points with correlation coefficients 0.74, 0.69, and 0.75, and (Monte Carlo permutation; *p*-values < 0.01).

**Fig. 7:**
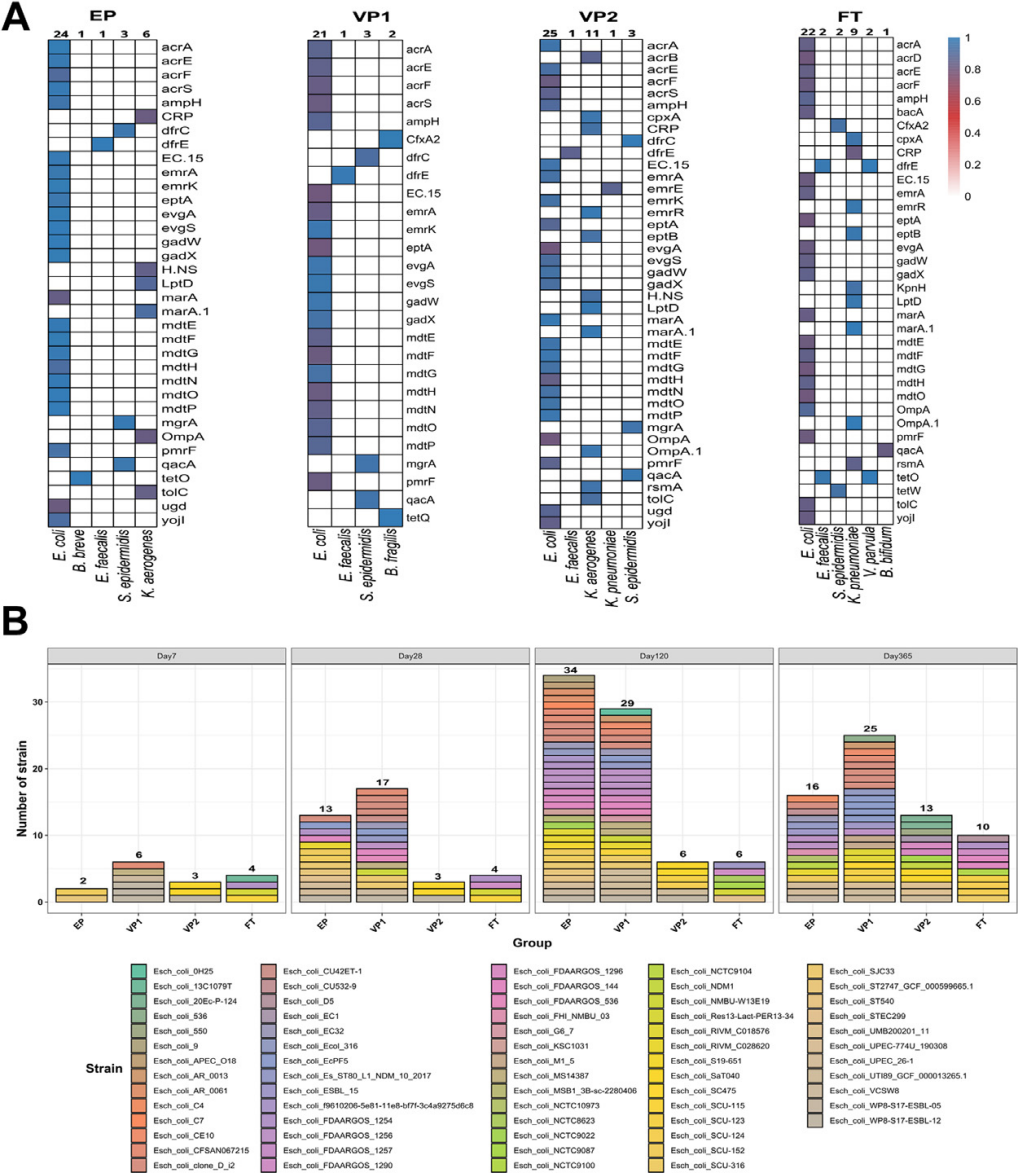
**The contribution of *E. coli* species to antibiotic resistance gene (ARG) load in infancy. (A)** Co-occurrence patterns between ARGs and microbial taxa on day 7 across the infant groups. The heatmap legend represents Spearman’s correlation coefficient (we excluded Spearman’s correlation <0.8). **(B)** The numbers of *E. coli* strains identified by StrainGE in the four infant groups through four time points: days 7, 28, 120, and 365. Abbreviations: extremely preterm infants (EP), very preterm infants (VP), and full-term infants (FT).

To predict the hosts of the identified ARGs across the infant groups, we searched for co-occurrence patterns between ARGs and microbial taxa on day 7 based on robust and significant correlations (Spearman’s rho  > 0.80, *p* < 0.01). *E. coli* was the potential host for 25 unique ARGs in the preterm groups and 22 ARGs in the full-term group (Fig. 7A). In addition, *K. aerogenes* in VP2 and *Klebsiella pneumoniae* in FT infants were the potential hosts for 11 and 9 ARGs, respectively.

*E. coli* relative abundance constituted the highest percentage among the potentially pathogenic taxa, and the above analysis predicted its association with the highest number of ARGs. Therefore, we characterised strain level dynamics of *E. coli* species in our cohort (Fig. 7B). Two unique *E. coli* strains were identified in the EP group on day 7. This number increased to around six-fold and 17-fold on days 28 and 120, respectively, before declining to 16 unique *E. coli* strains on day 365. We detected the same trend in the antibiotic-exposed VP1 group, although the numbers of *E. coli* strains were lower in the EP infants across all time points except day 120. The antibiotic-unexposed groups (VP2 and FT) had the lowest number of *E. coli* strains throughout the study, and their number remained constant at the first two time points before growing marginally at days 120 and 365. We also identified persistent *E. coli* strains in eleven infants, which were detected over at least three time points (Supplementary Fig. S11). The relative abundance of all persistent *E. coli* strains decreased over time, apart from *E. coli EcPF5* and *E. coli clone D i2* in infant number VP1-65, which increased slightly on day 120 before dropping to the same relative abundance on day 28.

## Discussion

In the current study, we used species-level metagenomic data and reported that probiotic supplementation in extremely preterm infants led to a dominance of the probiotic bacterium *B. longum* a few days after starting the intervention (Fig. 2). Contrarily, very preterm infants not supplemented with probiotics displayed a high prevalence of *E. coli*, *Staphylococcus epidermidis*, *E. faecalis*, and *K. aerogenes* in the first week of life. Such gut microbiota composition has been linked to an elevated risk of NEC and sepsis.^50^, ^51^, ^52^ These results also follow previous studies on the preterm gut microbiota, whose dynamics are driven by the species mentioned above that have pathogenic potential and are often multidrug-resistant.^7^^,^^53^ A significantly higher microbiota richness among term infants, compared to those born preterm, confirmed GA as a critical determinant of microbiota composition.^10^ The probiotic supplementation did not have any apparent effect on the gut microbiota richness, predicting that other factors connected to prematurity, such as intestinal epithelium immaturity, hospitalisation, and frequent antibiotic treatments, play a role.

Gut microbiota structure depends on the infant’s GA, putting extremely preterm infants at risk of delayed microbiota development.^10^ Fortification with natural early-life colonisers, such as *B. longum* subsp., *infantis* can beneficially modulate the preterm gut microbiota and lessen the detrimental influence of prolonged hospitalisation and antibiotic exposure.^19^ Our analyses based on statistical modelling of random ecological processes suggest that probiotics influence the transition to a more stable and mature microbial community as well as the complexity of the microbial interaction network (Fig. 4). This finding is in line with a randomised clinical trial of EP infants, which reported that the microbial community of probiotic-supplemented EP infants had a greater transition and stability probabilities to the most mature community type compared to the placebo group.^19^ According to random-forest modelling (Fig. 3D), probiotics were the third strongest predictor of microbiota development, supporting a link between the probiotic administration and their effect on gut microbiota maturation. In contrast, a longer duration of hospitalisation, which is closely correlated with decreasing GA, negatively impacted gut microbiota maturation, stability, and species interconnectivity, similar to findings from.^19^ We also found that the EP infants had the lowest predicted metabolic pathways diversity as compared to the other groups on day 7, indicating that extreme prematurity and associated treatments significantly altered the gut microbial functions in the first weeks of life (Supplementary Fig. S4B).

We detected antibiotic resistance genes in all infant samples, independent of prematurity or probiotic treatment. In the case of the antibiotic-unexposed VP infants, who displayed the highest ARG levels from all investigated infant groups on day 7, our study supports earlier evidence that antibiotic exposure throughout infancy is not essential for resistome development^7^^,^^27^ (Fig. 5A). Since preterm infants are exposed not only to antibiotics, the ARGs load might also be partly driven by non-antibiotic drugs promoting antibiotic resistance.^54^ In addition, faecal carriage of ARGs and multidrug resistance organisms in antibiotic-unexposed preterm infants have been attributed to transgenerational transmission, colonisation from environmental sources, or a survival-based response of the gut bacteria to antimicrobials produced by normal intestinal inhabitants.^27^ Finally, although antibiotic use might directly impact the ARGs profile of the gut microbiota, we did not find any association between the use of specific antibiotics and ARG profiles, similarly to other studies.^55^

We observed that probiotic supplementation was associated with reduced ARGs abundance in the EP infant gut in the first week of life, leading to a resistome carriage that was more similar to FT infants, akin to a recent report of very preterm infants given probiotics^30^ and our previous analysis.^21^ Moreover, our study design, which comprised different GA groups and varying lengths of hospital stay, demonstrated that hospitalisation impacts the resistome composition. This result confirms a previous study documenting that hospitalisation enriched the gut antibiotic resistome in preterm infants.^7^ We also found that the ARGs load in VP newborns was higher in the first few weeks after birth and gradually decreased over the first year of life, owing to a decrease in the abundance of efflux pumps, similarly to previous research.^31^^,^^56^

Despite the significant differences in the resistome composition at the first week of life, the relative abundance of the identified ARGs in the EP infants was not significantly different from that of more mature infants at 1, 4, and 12 months of age (Fig. 5C), a finding we reported earlier with the use of different bioinformatics tools.^21^ Although not directly comparable because of different microbiota composition and richness between infants and adults, recent reports investigating the adult population suggested that supplementation with probiotic strains exacerbates resistome expansion in the gut mucosa.^57^^,^^58^ However, we observed no significant differences in the alpha diversity of ARGs, despite the Chao1 and Shannon diversity indices indicating that FT infants had the lowest ARG diversity (Supplementary Fig. S8). This finding contradicts a recent study that reported a significant decrease in the diversity of ARGs in all probiotic-supplemented preterm infants as compared to other preterm (<32 weeks) and full-term infants.^30^ This discrepancy can be caused by using a targeted enrichment for ARGs in the latter mentioned study, as compared to the shotgun metagenomic sequencing applied to samples from our cohort. Additionally, we observed no significant influence of birth mode on the resistome as well as on the gut microbiota (Supplementary Fig. S3 and S6). Full-term infants delivered via Caesarean section have been reported to have a higher relative abundance of ARGs compared to vaginally-delivered infants,^29^^,^^31^ however, the mode of delivery does not seem to have the same impact on the gut microbiota or resistome of preterm infants as in term-born infants.^59^

The probiotic-supplemented EP infants had a significantly higher abundance of MGEs than VP and FT infants (Fig. 6). Moreover, Shannon diversity indices revealed that EP infants had the highest MGEs diversity across the study. Compared to FT, the significantly higher mobilome carriage in EP infants indicates a persistent effect of extreme prematurity and associated external factors on the gut mobilome. Theoretically, the MGEs that we identified in the probiotic strains (Supplementary Fig. S9) could mediate the horizontal gene transfer of other genes from the probiotic strains^60^ to other bacteria taxa and increase the likelihood that ARGs from other sources will be mobilised. However, the likelihood of such events appears to be low, as the probiotic-derived MGEs represented about 0.75% of all identified MGEs. Nonetheless, other factors, such as prolonged hospitalisation and associated antibiotic use, immaturity of the intestinal tract, transgenerational transmission, and preterm gut microbiota, are likely to contribute to the total MGEs load. More investigations are needed to comprehend early life resistome and mobilome dynamics and whether they colocalise within the same gut bacterial taxa.

The observed early-life resistome profiles correlated with the taxonomic composition of the gut microbiota across the infant groups (Fig. 7). This result corroborates earlier evidence showing that the relative abundances of certain ARGs typically rise promptly after antibiotic treatment and decline shortly after cessation.^7^^,^^27^^,^^31^ Such spikes are strongly connected with commensurate shifts in the abundance of specific species. The Procrustes analysis also identified that the early life mobilome correlated to the gut microbiota’s taxonomic composition on day 7, suggesting that community structure shaped the mobilome composition. Additionally, mobilome and resistome were robustly correlated and remained so for all measured timepoints.

*E. coli* is a major contributor to the increased ARGs load in infancy,^7^^,^^27^ and its abundance robustly correlated with infants’ resistome (Fig. 7A). A majority of identified ARGs were chromosomal genes, e.g., *mdtE* and *marA*, which may partially explain the strong correlations between specific ARGs and species. Recent research reported that *E. coli* is the potential bacterial host of 36 of the 50 most prevalent ARGs in the newborn microbiota.^31^ Our study identified that *K. pneumoniae* and *K. aerogenes* also likely contribute to the ARGs load in term infants. This finding agrees with a recent meta-analysis that revealed intestinal microbiota dominated by *Klebsiella* spp., has comparably high relative ARG abundances,^28^ highlighting that *E. coli* is not the only predictor of increased resistome burden in early life.

One of the main limitations of our study is the lack of a GA-matched group of EP infants not receiving probiotic supplementation. We could not recruit such a group as all EP infants in Norway during the study period received probiotics as a standard of care. Processing samples in two sequencing runs was also not optimal due to the possibility of batch variations. However, in our assessment of confounder status, we did not detect the two different sequencing batches as covariates with significant impact on the gut microbiota composition (Supplementary Fig. S1).

The NICU environment has been shown as a variable in shaping the infants’ gut microbiota^18^; however, we did not study the related aspects, including the microbiota of health personnel, feeding tubes, and hospital surfaces. Similarly, the maternal gut microbiome, breast milk microbiome, and maternal antibiotic exposure history during pregnancy have been associated with infant gut microbiota and resistome,^27^ but it was beyond the scope of the current clinical study to include collection and analyses of maternal samples/data.

Diet has a pivotal role in shaping the infants’ gut microbiota and resistome,^27^^,^^28^ and most infants were fed human breastmilk for the first two timepoints (Supplementary Table S2), which is the standard for preterm infants in Norwegian NICUs and for healthy newborns in Norway. However, we could not perform rigorous statistical analysis evaluating the impact of different feeding practices on gut microbiota composition, resistome, and mobilome over time due to missing dietary data for the later time points (Supplementary Table S2). Similarly, we had a limited data on the infants hospitalisation between 4th and 12th month of life, which indicated a similar trend on lower respiratory tract infections in preterm infants as reported earlier.^61^ Due to these missing data, we could not explore whether there exist any associations with the infant gut microbiota or resistome.

In addition, all preterm infants (both extremely and very preterm) in this study were fed by a nasogastric or orogastric tube, a factor that has been recently shown to have a considerable impact on bacterial colonization^15^ and ARGs transfer.^62^ As we did not assess the microbial composition of the feeding tubes, we cannot exclude their impact on the preterm infants’ gut microbiota and resistome. Finally, the lack of data on non-antibiotic drug exposure at NICUs and antibiotic use from 4 up to 12 months of life hindered us from analysing associations between these covariates and early-life microbiota, resistome, and mobilome.

Overall, our study indicates the microbiological benefit of probiotic supplementation to extremely preterm infants in the NICUs to alleviate the harmful effects of antibiotics and hospitalisation on gut microbiota composition. Probiotic administration aided the microbiota development by promoting microbial community interconnectivity and stability in the first week of life and minimised resistome development induced by antibiotic usage and hospitalisation. For the first time, we describe the dynamics of mobilome development in infants across varying gestational ages, antibiotic exposure, and probiotic supplementation. This novel analysis of MGE carriage and distribution was neither performed in the earlier study of the same cohort nor any other infant cohort to date. Finally, we identified the relative abundance of *E. coli* and *K. pneumoniae* in antibiotic-exposed infants as predictors of increased resistome burden in early life. Still, to provide causative evidence for our findings, additional experimental studies are needed, such as testing the effect of altered microbiota in gnotobiotic mice, and detection of MGEs and ARGs in *E. coli* and *K. pneumoniae* clinical isolates. Similarly, additional longitudinal multi-centre studies, based on deep metagenome sequencing and augmented with functional metagenomics and metabolomics, are essential to further inform guidelines on probiotic supplementation in the NICUs.

## Contributors

A.B. performed the bioinformatical and statistical analyses and wrote the initial draft of the manuscript. C.K. conceptualised and designed the study, secured funding, contributed during data analyses, and oversaw all aspects of the research. E.E. participated in the study design, clinical data collection, and laboratory work. J.P.C. and E.H. were involved in the study design and discussions on the bioinformatics analyses. J.B.P. advised on the data analyses and revised the manuscript for intellectual content. V.K.P. supervised the data analyses and manuscript preparation. V.K.P., C.K., and A.B. verified the underlying data. All authors read and approved the final version of the manuscript.

## Data sharing statement

Upon filtering out the human DNA, shotgun metagenomic reads have been deposited to SRA under BioProject ID PRJNA898628 (Supplementary Table S4). The software packages used in this study are open-source and free. The database for taxonomic profiling is the mpa_v30_CHOCOPhlAn_201901 database (https://zenodo.org/record/3955744#.Yr2AwHZBy38). Human genome reference, GRCh38 (https://hgdownload.soe.ucsc.edu/goldenPath/hg38/bigZips/). The databases for the functional profiling (ChocoPhlAn and full UniRef90) were downloaded by the command line suggested in the HUMAnN manual. CARD v.3.0.9 (https://card.mcmaster.ca/download). Database of MGES curated by NanoARG v.1.0 (https://bench.cs.vt.edu/ftp/data/databases/).

## Declaration of interests

The authors have declared that no competing interest exists.

## Appendix A Supplementary data

Supplementary Tables S1–S5

STORMS Checklist
