# Supplementary material for: Development of early life gut resistome and mobilome across gestational ages and microbiota-modifying treatments
Source: eBioMedicine. 2023 May 13;92:104613. doi: 10.1016/j.ebiom.2023.104613 (PMC10192547; doi:10.1016/j.ebiom.2023.104613)
Supplement: Supplementary Figs. S1–S11 [file mmc3.docx]

**Supplementary Figures for**

Development of early life gut resistome and mobilome across gestational ages and microbiota-modifying treatments

By Bargheet et al.

**Supplementary fig. S1:** Identification of cofounders that influence the abundance of the microbiota**.**

**Supplementary fig. S2:** The relative abundances of bacterial genera.

**Supplementary fig. S3:** The relative abundances of bacterial genera and species, and Shannon index across the infant groups classified by birth mode.

**Supplementary fig. S4:** Predicted metabolic pathways of the gut microbiota.

**Supplementary fig. S5:** Genus-level composition of five microbiota community types (MC) estimated by Dirichlet Multinomial Mixture (DMM).

**Supplementary fig. S6:** The relative abundance of the antibiotic resistant genes (ARGs) across the infant groups classified by birth mode.

**Supplementary fig. S7:** The relative abundances of antibiotic resistance genes classes across infant groups, as estimated by DESeq2.

**Supplementary fig. S8:** Antibiotic-resistant genes richness (Chao1) and evenness (Shannon) indices comparison between the infant groups.

**Supplementary fig. S9:** MGEs detected in the Infloran® *Bifidobacterium longum* subspecies infantis and *Lactobacillus acidophilus*, the probiotic given to the EP infants.

**Supplementary fig. S10:** Correlations between the gut microbiota, antibiotic resistance genes (ARGs), and mobile genetic elements (MGEs).

**Supplementary fig. S11:** The relative abundance of the persistent *E. coli* strains identified by StrainGE of four infant groups (EP, VP1, VP2, and FT) through at least three time points.

#
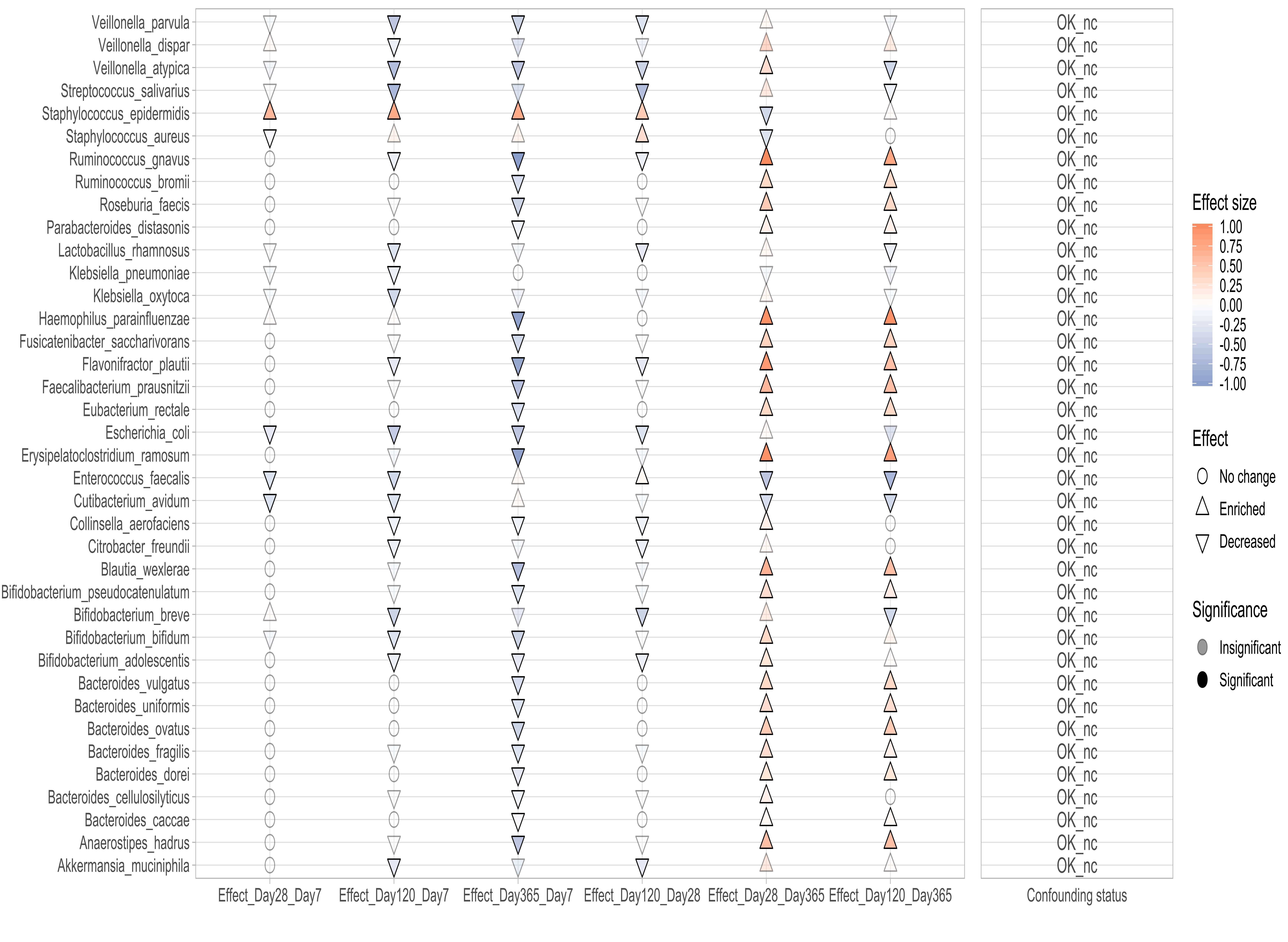


Fig. S1 | **Identification of cofounders that influence the abundance of the microbiota.** An analysis of the cofounder status identified an influence of antibiotic treatment on the gut microbiota abundance. However, no other potential covariate was identified as significant, confirming that a sequencing batch effect had no significant impact on the bacterial species composition.

#
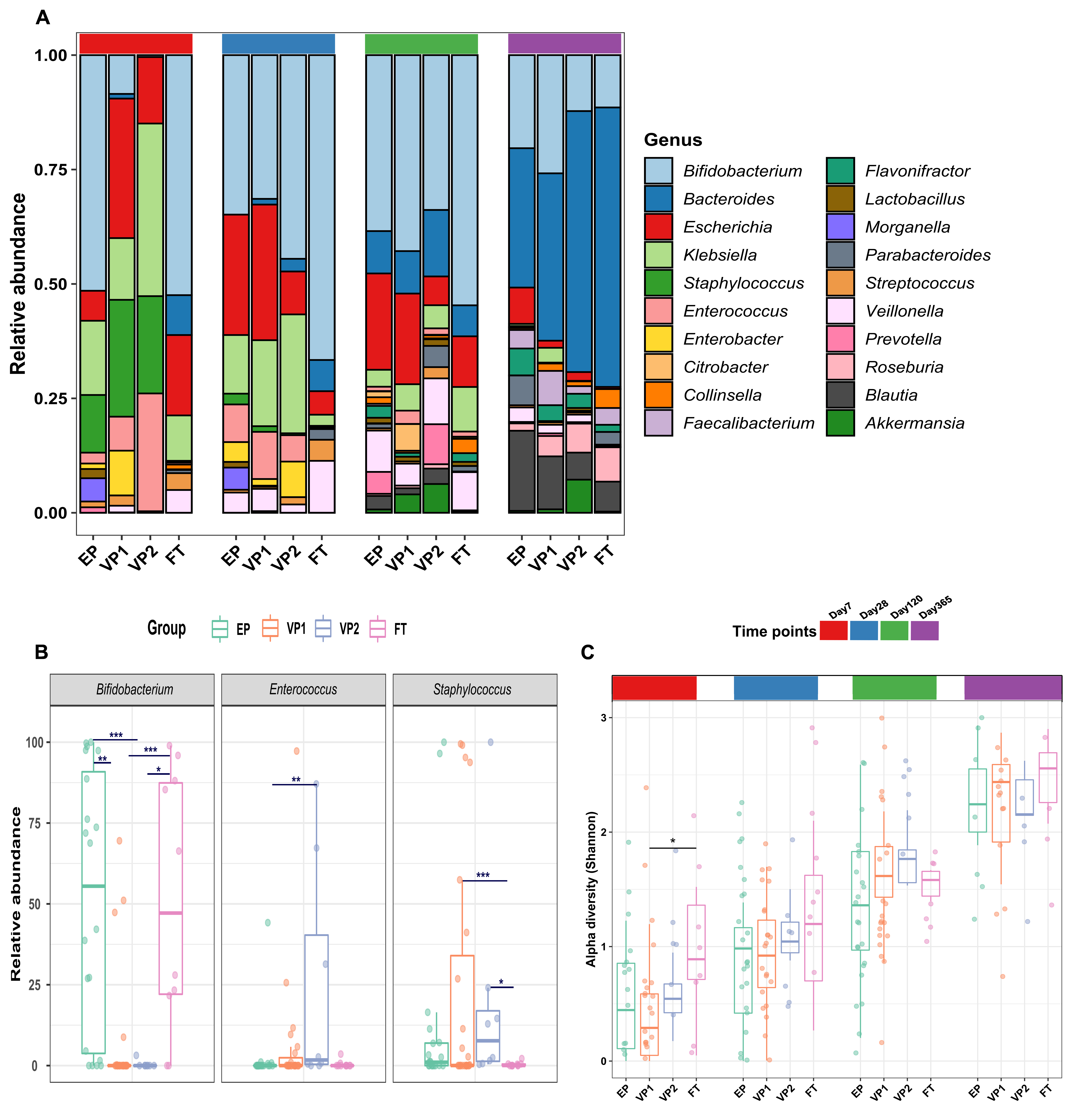


Fig. S2 | A) **The relative abundances of bacterial genera inferred by MetaPhlAn3 for 4 infant groups** (extremely preterm EP - antibiotic-exposed and probiotic supplemented; very preterm VP1 - antibiotic-exposed and VP2 - antibiotic unexposed; full-term infants FT - antibiotic unexposed) and four time points. B) **The relative abundances of microbiota on day 7 at the genus level showed a significant difference across groups estimated by DESeq2**. The *p* values were computed using the Wald test. Adjusted *p* values (adj *p*): ^***^adj *p* < 0.001; ^**^adj *p* < 0.01; ^*^adj *p* < 0.05. C) **Shannon diversity comparison between the groups**. Each point represents a sample. The horizontal box lines represent the first quartile, the median, and the third Quartile. The *p* values were computed using the One-way ANOVA and adjusted using Tukey’s HSD post hoc test. Adjusted *p* values (adj *p*): ^*^adj *p* < 0.05.


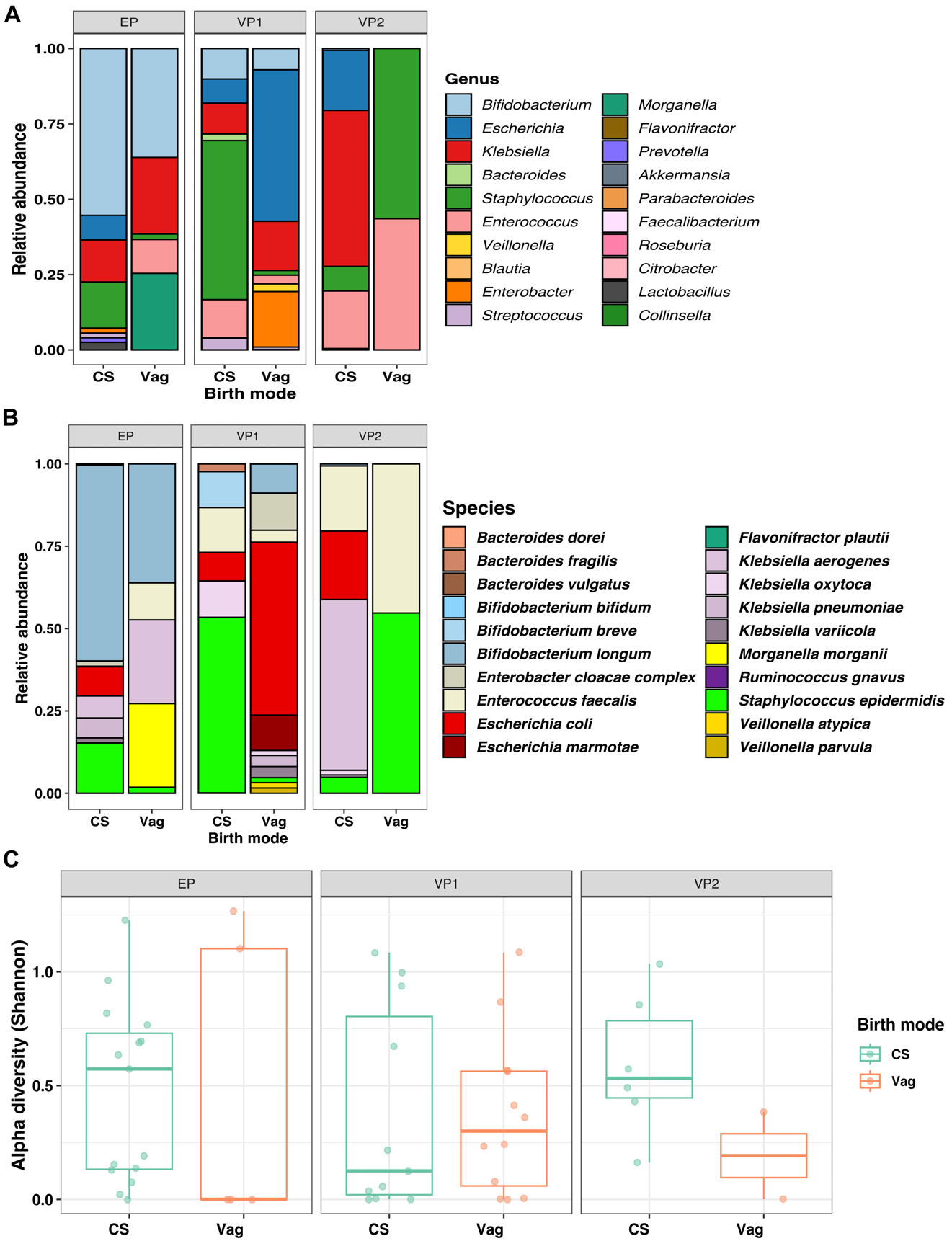


Fig. S3 | **The relative abundances of bacterial genera** (A) **and species** (B) **inferred by MetaPhlAn3 for 3 infant groups** (EP; C-section *n* = 15 vs Vaginally *n* = 5, VP1; C-section *n* = 12 vs Vaginally *n* = 11, VP2; C-section *n* = 6 vs Vaginally *n* = 2) **classified by birth mode.** C) **Shannon index across the infant groups classified by birth mode**. We found no significant difference between groups. Each point represents a sample. The horizontal box lines represent the first quartile, the median, and the third Quartile. Abbreviations: extremely preterm infants (EP), very preterm infants (VP), and full-term infants (FT), Caesarean section (CS), Vaginal delivery (Vag).


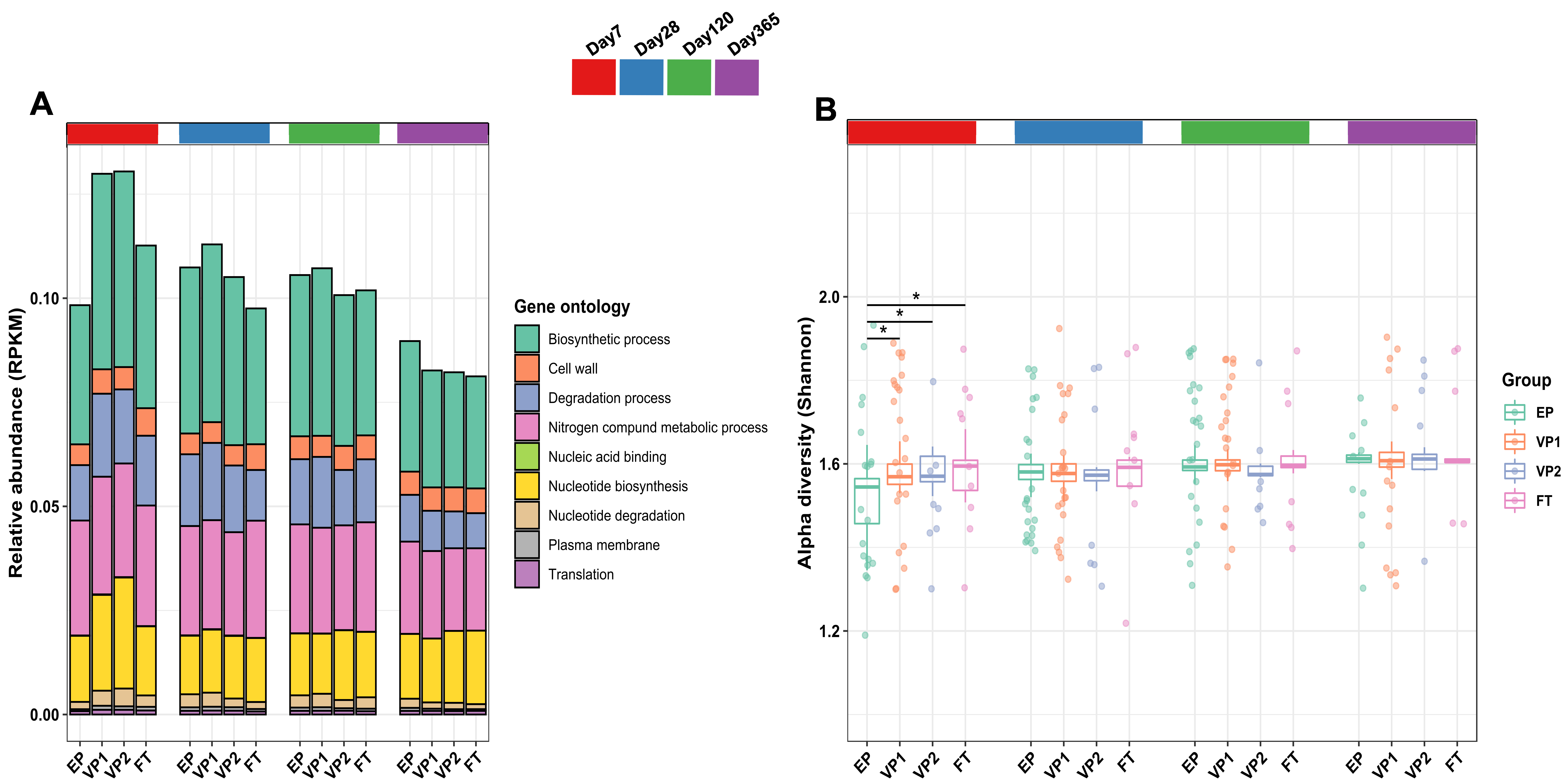


Fig. S4 | **Predicted metabolic pathways of the gut microbiota**. (A) HUMAnN3 identified the relative abundances of the microbiota functional compositions for all neonates included in the study. (B) Shannon diversity of metabolic pathways comparison between the groups. Each point corresponds to a given sample. The horizontal box lines represent the first quartile, the median, and the third Quartile. The *p* values were computed using the One-way ANOVA and adjusted using Tukey’s HSD post hoc test. Adjusted *p* values (adj *p*): ^*^adj *p* < 0.05. Abbreviations: extremely preterm infants (EP), very preterm infants (VP), and full-term infants (FT).

**Group**





Fig. S5 | **Genus-level composition of five microbiota community types (MC) estimated by Dirichlet Multinomial Mixture (DMM).**

**
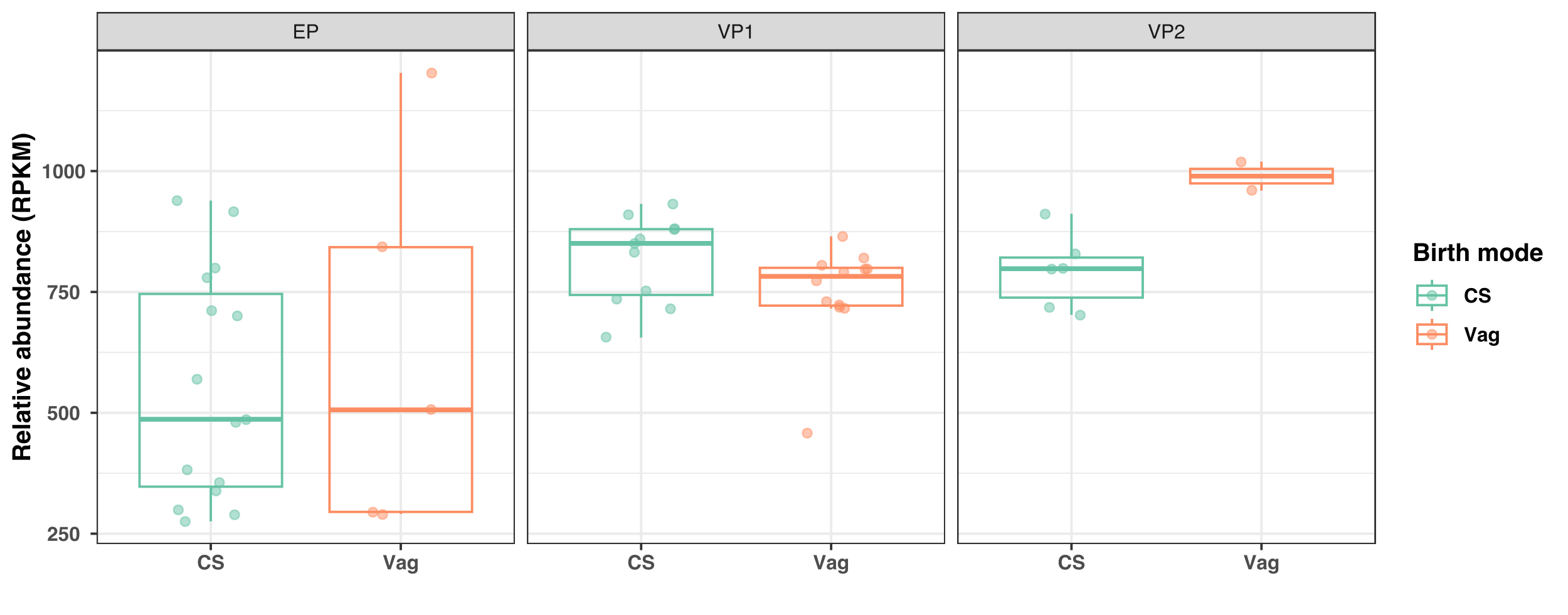
**

Fig. S6 | **The relative abundance of the antibiotic resistant genes (ARGs) across the infant groups classified by birth mode**. Each point represents a sample. The horizontal box lines represent the first quartile, the median, and the third Quartile. Abbreviations: extremely preterm infants (EP), very preterm infants (VP), and full-term infants (FT), Caesarean section (CS), Vaginal delivery (Vag).


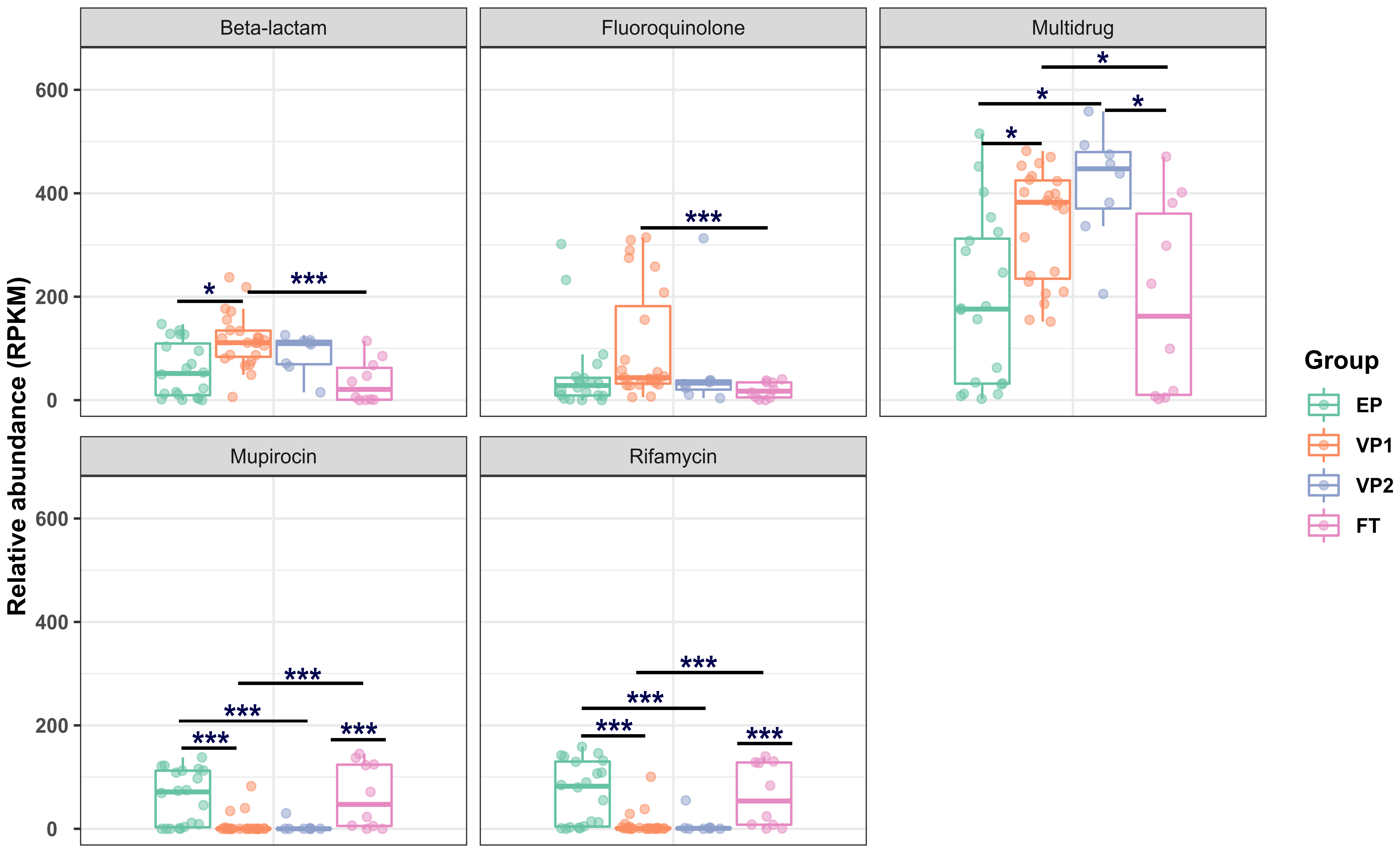


Fig. S7 | **The relative abundances of antibiotic resistance genes classes across infant groups, as estimated by DESeq2**. The horizontal box lines represent the first quartile, the median, and the third Quartile. The *p* values were computed using the Wald test. Adjusted *p* values (adj *p*): ^***^adj *p* < 0.001; ^**^adj *p* < 0.01; ^*^adj *p* < 0.05. Abbreviations: extremely preterm infants (EP), very preterm infants (VP), and full-term infants (FT).


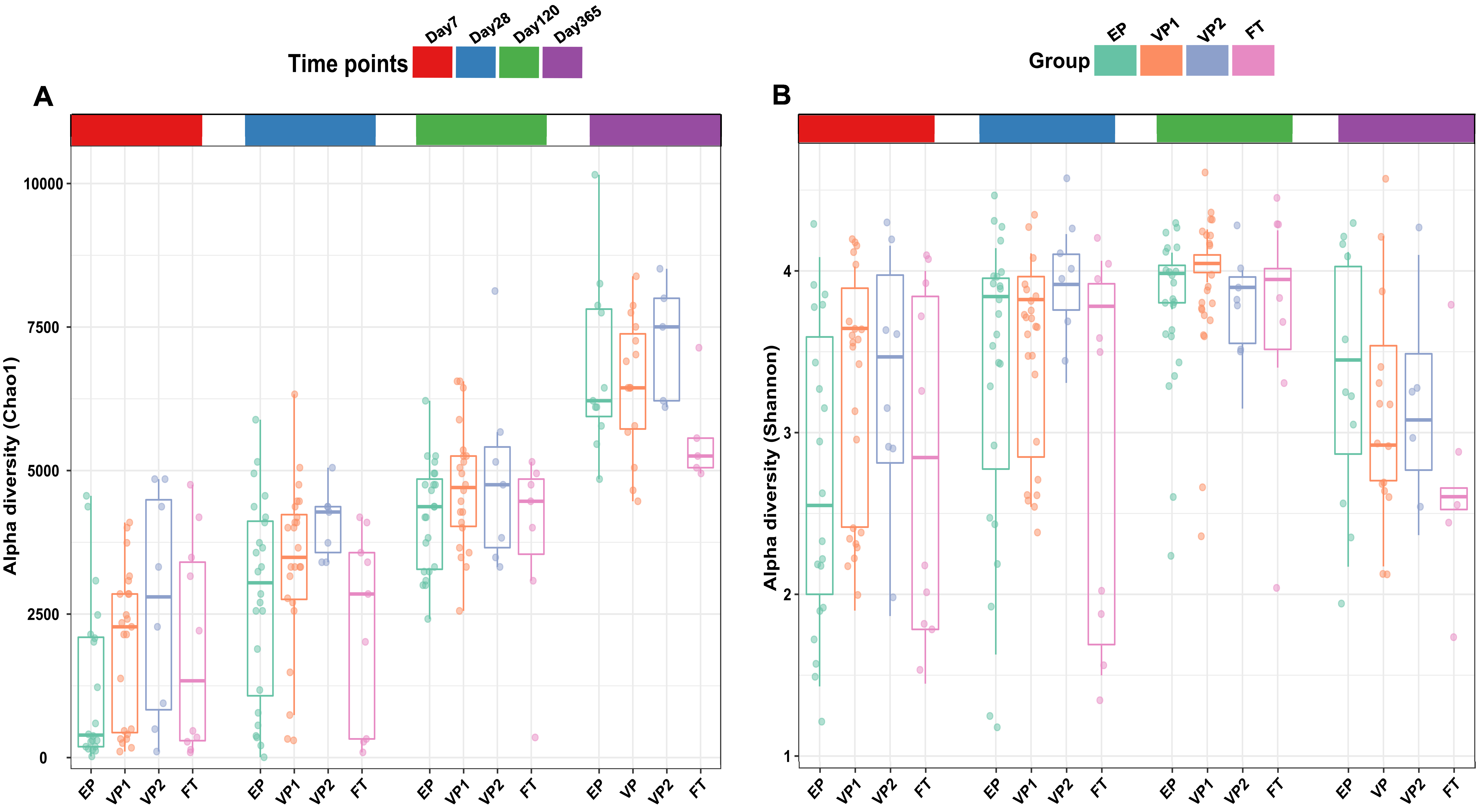


Fig. S8 | **Antibiotic resistant genes (ARGs) richness (Chao1) (A) and evenness (Shannon) (B) indices comparison between the infant groups**. Each point represents a sample. The horizontal box lines represent the first quartile, the median, and the third Quartile. Abbreviations: extremely preterm infants (EP), very preterm infants (VP), and full-term infants (FT).

**

**

Fig. S9 | (A) **MGEs detected in the Infloran® *Bifidobacterium longum* subspecies *infantis* and *Lactobacillus* *acidophilus*, the probiotic given to the EP infants**. (**B**) The percentage of MGEs classes detected in the Infloran®.

**
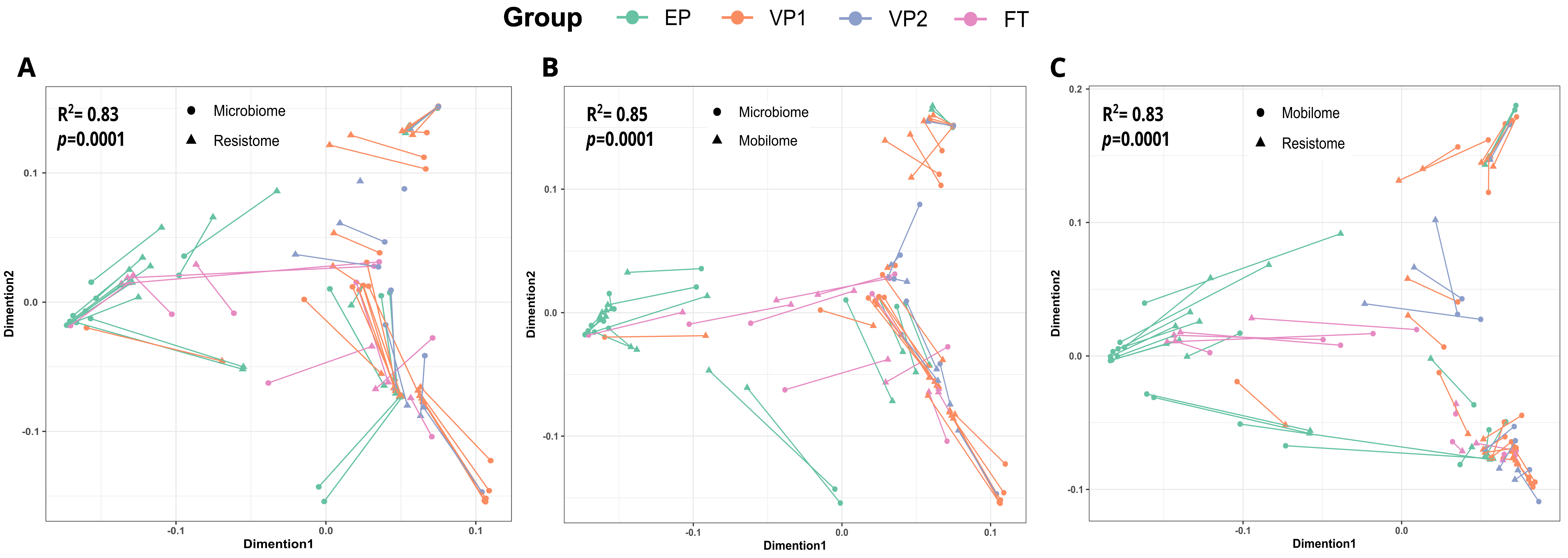
**

Fig. S10 | **Correlations between the gut microbiota, antibiotic resistance genes (ARGs), and mobile genetic elements (MGEs)**. Procrustes analysis of the correlation between **(A)** taxonomic composition and ARGs, **(B)** taxonomic composition and MGEs, and **(C)** MGEs and ARGs on day 7. The line length linking the two points represents the degree of dissimilarity between the two distinct compositions of the same sample. Abbreviations: extremely preterm infants (EP), very preterm infants (VP), and full-term infants (FT).

**
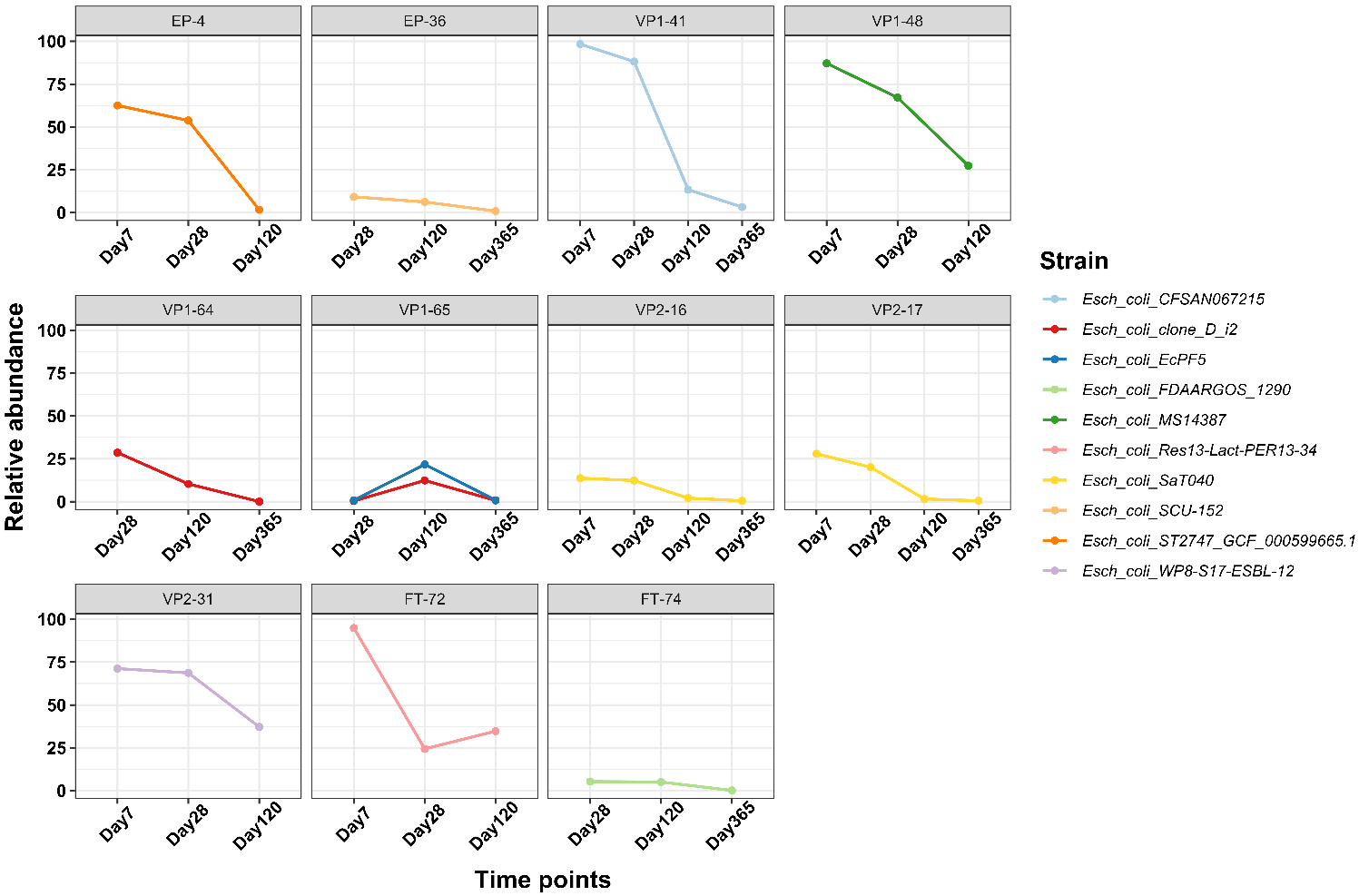
**

Fig. S11 | **The relative abundance of the persistent *E. coli* strains identified by StrainGE of four infant groups (EP, VP1, VP2, and FT) through at least three time points.** The number beside the group name refers to anonymised individual identification number. Abbreviations: extremely preterm infants (EP), very preterm infants (VP), and full-term infants (FT).
